# Association of first primary cancer with risk of subsequent primary cancer among survivors of adult-onset cancers in Kentucky and Appalachian Kentucky

**DOI:** 10.3389/fonc.2023.1193487

**Published:** 2023-08-17

**Authors:** Quan Chen, Bin Huang, Abigail M. Anderson, Eric B. Durbin, Susanne M. Arnold, Jill M. Kolesar

**Affiliations:** ^1^ Markey Cancer Center, University of Kentucky, Lexington, KY, United States; ^2^ Division of Cancer Biostatistics, University of Kentucky, Lexington, KY, United States; ^3^ Department of Radiology, University of Kentucky, Lexington, KY, United States; ^4^ Department of Internal Medicine, University of Kentucky, Lexington, KY, United States; ^5^ Department of Obstetrics and Gynecology, University of Kentucky, Lexington, KY, United States; ^6^ Department of Pharmacy Practice and Science, University of Kentucky, Lexington, KY, United States

**Keywords:** subsequent primary cancer, first primary cancer, cancer survivors, Appalachian Kentucky, surveillance, cancer disparities, cancer associated risk factors

## Abstract

**Background:**

Appalachia is a region with significant cancer disparities in incidence and mortality compared to Kentucky and the United States. However, the contribution of these cancer health disparities to subsequent primary cancers (SPCs) among survivors of adult-onset cancers is limited. This study aimed to quantify the overall and cancer type-specific risks of SPCs among adult-onset cancer survivors by first primary cancer (FPC) types, residence and sex.

**Methods:**

This retrospective cohort study from the Kentucky Cancer Registry included 148,509 individuals aged 20-84 years diagnosed with FPCs from 2000-2014 (followed until December 31, 2019) and survived at least 5 years. Expected numbers of SPC were derived from incidence rates in the Kentucky population; standardized incidence ratio (SIR) compared with those expected in the general Kentucky population.

**Results:**

Among 148,509 survivors (50.2% women, 27.9% Appalachian), 17,970 SPC cases occurred during 829,530 person-years of follow-up (mean, 5.6 years). Among men, the overall risk of developing any SPCs was statistically significantly higher for 20 of the 30 FPC types, as compared with risks in the general population. Among women, the overall risk of developing any SPCs was statistically significantly higher for 20 of the 31 FPC types, as compared to the general population. The highest overall SIR were estimated among oral cancer survivors (SIR, 2.14 [95% CI, 1.97-2.33] among men, and among laryngeal cancer survivors (SIR, 3.62 [95% CI, 2.93-4.42], among women. Appalachian survivors had significantly increased risk of overall SPC and different site specific SPC when compared to non-Appalachian survivors. The highest overall SIR were estimated among laryngeal cancer survivors for both Appalachian and non-Appalachian residents (SIR, 2.50: 95%CI, 2.10-2.95; SIR, 2.02: 95% CI, 1.77-2.03, respectively).

**Conclusion:**

Among adult-onset cancer survivors in Kentucky, several FPC types were significantly associated with greater risk of developing an SPC, compared with the general population. Risk for Appalachian survivors was even higher when compared to non-Appalachian residents, but was not explained by higher risk of smoking related cancers. Cancers associated with smoking comprised substantial proportions of overall SPC incidence among all survivors and highlight the importance of ongoing surveillance and efforts to prevent new cancers among survivors.

## 1 Introduction

The United States (US) 5-year survival rate for all cancers increased from 49% to 68% between the mid-1970s to 2011 and 2017 (1). In 2022, there were an estimated 18 million cancer survivors in the US (2) and the number is projected to continue to increase to 22.1 million by the year 2030 (3). Cancer survivors are at risk for the development of new malignancies, or subsequent primary cancers (SPCs) diagnosed after their first primary cancer (FPC) (4). Nationally, SPCs account for approximately 25% of newly diagnosed cancers in patients 65 years or older and 11% for adults younger than 65 (5). Older age at diagnosis and a modifiable risk factor [e.g.; smoking, obesity, alcohol or infection related (6–9)] associated FPC both increase the risk of developing SPCs (10–12). Sung and colleagues recently reported an analysis of twelve Surveillance, Epidemiology, and End Results (SEER) registries in the United States assessing the risk of SPCs in individuals diagnosed with a FPC and surviving 5 years, demonstrating that risk of death due to SPCs was higher among cancer survivors than the general population and that cancers associated with modifiable risk factors, like smoking and obesity, comprise a significant proportion of SPCs (11).

While cancer incidence and mortality rates have steadily declined in the U.S., rural Appalachia Kentucky is an exception, where incidence continues to increase, mortality rates are 36% higher than urban non-Appalachians and 5 year survival is only 50% (13). In addition, Kentucky leads the nation in both lung cancer incidence and mortality. Excess cancer incidence and mortality is typically attributed to high rates of cancer associated risk factors, however a recent cross-sectional study demonstrated that the impact of risk factors on cancer mortality varies by region. In the South, smoking, receipt of Supplemental Nutrition Assistance Program (SNAP) (a surrogate indicator of low income) benefits and physical inactivity were over 60% relative importance. In Appalachia receiving SNAP benefits was of high importance and in Kentucky, smoking was of high importance (14). Interestingly, risk factor importance did not correspond completely with risk factor prevalence, which suggests the potential of unobserved and location specific risk factors for further exploration.

Given Kentucky’s high incidence and poor survival related to cancers, significant disparities and the presence of region-specific cancer risk factors, the objective of this study was to determine the frequency of SPCs in patients diagnosed with cancer in Kentucky and Appalachian Kentucky.

## 2 Materials and methods

### 2.1 Study population and first primary cancer

Cancer cases diagnosed at ages 20 to 84 during 2000-2014 in the Kentucky cancer registry were selected from SEER 17 research plus 2021 November submission data (15). FPC sites were categorized based on the *International Classification of Disease for Oncology Third Edition (ICD-O-3)-World Health Organization 2008 definition* (16). Patients with at least five years of survival were included to estimate the long-term survivorships and permit comparison of findings with published results (11).

### 2.2 Subsequent primary cancer cases ascertainment

All new SPCs were included based on the SEER’s rule for multiple primaries, except non-melanoma skin cancer (17). The follow-up of SPC began after five years of their FPC diagnosis, a 60-months latency exclusion, and continued until death or loss to follow-up, attained age 90 or older, or at the study cut-off time (December 31, 2019), whichever came first. Multiple events of SPC were allowed so the survivor could continue to contribute person-time at risk for their entire time in the study (18).

### 2.3 Analytical variables

Variables included in this study include cancer survivors’ age categorized into four groups: 20-49, 50-64, 65-74, and 75 years older. The race was coded as white, black, other, and unknown based on the SEER race recode group (19). Only patients with male or female sex were included. The years of FPC diagnosis were grouped in five year increments. SEER historic stages were selected for consistency of stage comparison due to the change of stage definitions over the years (20). According to the Appalachia Regional Commission, Appalachian regions, especially Kentucky Appalachia, suffer more significant economic and cancer burdens than non-Appalachia regions. The geographical variable indicated whether patients’ residences were in Appalachia or not at the time the cancer diagnosis were created (21).

### 2.4 Statistical analysis

The estimates of SPC were compared with the expected incidence in the general Kentucky population by calculating standardized incidence ratios (SIRs) and 95% confidence intervals (CI). SIR was calculated as the ratio of the observed to the expected number of SPCs (22). The expected numbers of SPC were derived from Kentucky incidence rates in the SEER incidence file 2021 submission, stratified by age (5-year groups), race (white/unknown, black, other), sex, and calendar year (5-year groups), multiplied by the appropriate person-years at risk. Statistics of patients’ SPC on any site, same site, and sites other than the FPC were estimated using the SIRs and 95% CI. Results were further stratified by sex and Appalachian region to compare site-specific risks of developing SPC. Cancer sites with 200 or more survivors in the full cohort were presented for site-specific risk assessment in the Tables and Figures.

To further understand the impacts of risk factors on SPC, the standard definitions for risk factor-associated cancers were adopted from the Centers for Disease Control and Prevention (CDC) (23). The SIRs were estimated to assess the risk of developing any risk factor associated with SPC within the same category of FPC for 12 tobacco-associated cancers [oropharynx, esophagus, stomach, colorectal, liver, pancreas, larynx, lung, cervix, kidney, urinary bladder, acute myeloid leukemia], 13 obesity-associated cancers [esophageal adenocarcinoma, gastric cardia, colorectal, liver, gallbladder, pancreas, multiple myeloma, postmenopausal female breast, corpus and uterus, ovary, kidney, meningioma, thyroid], six alcohol-associated cancers [oropharynx, esophagus, colorectal, liver, larynx, female breast], three physical inactivity-associated cancers [colon, postmenopausal female breast, corpus and uterus, NOS], and six Human Papillomavirus (HPV)-associated cancers [oropharyngeal squamous cell carcinoma, anal and rectal squamous cell carcinoma, vulvar squamous cell carcinoma, vaginal squamous cell carcinoma, cervical carcinoma, penile squamous cell carcinoma]. All statistical tests were two-sided with a significance level of p < 0.05. Analysis was conducted utilizing SAS 9.4(Cary, NC) and SEER*Stat 8.4.0.

### 2.5 Ethical considerations

This study was approved by the University of Kentucky Institutional Review Board (IRB # 78447). All data were treated as confidential and only accessible in password-protected files for authorized study staff.

## 3 Results

Between 2000-2014, 148,509 patients in KY diagnosed with an invasive FPC who survived at least 5 years were included in the study (**Table 1**). Women (51%, 74,508) and men (49%, 74,001) were approximately equally represented. The majority of patients were white (93%, 138,178) and resided in non-Appalachian regions (72%, 107,121). Participants lived a mean of 5.59 years after SPC and longer than 10 years after FPC diagnosis, accruing 829,530 person-years of follow-up.

**Table 1:** Characteristics of patients diagnosed with Invasive FPC in 2000-2014, KY, 20-84, with >= 5 years of survival.

	Survivors	# survivors without SPC		# survivors with only 1 SPC*		# survivors with more than 2 SPC*		Total Observed SPC	Person Years at Risk	Mean Person Years at Risk
**Total**	148,509	131,978	88.9%	14,931	10.1%	1,600	1.1%	17970	829,530.28	5.59
Sex										
Male	74,001	64,910	87.7%	8,154	11.0%	937	1.3%	9958	404,696.27	5.47
Female	74,508	67,068	90.0%	6,777	9.1%	663	0.9%	8012	424,834.01	5.7
Age of FPC										
20-49	31,318	29,145	93.1%	1,983	6.3%	190	0.6%	2382	210,658.92	6.73
50-64	60,956	53,748	88.2%	6,443	10.6%	765	1.3%	8068	360,944.45	5.92
65-74	38,947	33,664	86.4%	4,778	12.3%	505	1.3%	5822	200,830.68	5.16
75+	17,288	15,421	89.2%	1,727	10.0%	140	0.8%	1698	57,096.22	3.3
FPC Diagnosis Year										
2000-04	45,215	37,152	82.2%	7,141	15.8%	922	2.0%	8901	400,628.73	8.86
2005-09	50,820	44,765	88.1%	5,516	10.9%	539	1.1%	6532	307,363.19	6.05
2010-14	52,474	50,061	95.4%	2,274	4.3%	139	0.3%	2537	121,538.36	2.32
Months of follow up since FPC										
60-119	74,431	68,178	91.6%	5,885	7.9%	368	0.5%	6550	173,781.73	2.33
120-179	48,016	41,704	86.9%	5,582	11.6%	730	1.5%	6943	342,518.92	7.13
180+	26,062	22,096	84.8%	3,464	13.3%	502	1.9%	4477	313,229.63	12.02
Race										
White	138,178	122,648	88.8%	14,036	10.2%	1,494	1.1%	16877	772,997.35	5.59
Black	8,805	7,854	89.2%	848	9.6%	103	1.2%	1040	48,090.26	5.46
Other	636	596	93.7%	37	5.8%	3	0.5%	43	3,284.02	5.16
Unknown	890	880	98.9%	10	1.1%	0	0.0%	10	5,158.65	5.8
SEER Historic Stage A										
Localized	68,423	60,714	88.7%	6,932	10.1%	777	1.1%	8425	392,678.51	5.74
Regional	25,639	22,757	88.8%	2,615	10.2%	267	1.0%	3128	137,281.17	5.35
Distant	8,686	7,864	90.5%	754	8.7%	68	0.8%	878	37,964.13	4.37
Localized/Regional (Prostate cases)	28,982	25,839	89.2%	2,845	9.8%	298	1.0%	3389	167,977.67	5.8
Unstaged	15,135	13,421	88.7%	1,548	10.2%	166	1.1%	1861	86,982.36	5.75
Blank(s)	1,644	1,383	84.1%	237	14.4%	24	1.5%	289	6,646.44	4.04
Appalachia Status										
Not Appalachia	107,121	95,313	89.0%	10,661	10.0%	1,147	1.1%	12833	601,792.11	5.62
Appalachia	41,388	36,665	88.6%	4,270	10.3%	453	1.1%	5137	227,738.17	5.5
Reason for exit from the study										
Alive	105,175	97,743	92.9%	6734	6.4%	698	0.7%	8144	644,709.71	6.13
Lost to Follow-up	3,582	3,469	96.8%	105	2.9%	8	0.2%			
End of Study	101,593	94,274	92.8%	6,629	6.5%	690	0.7%			
Death	43,334	34,235	79.0%	8,197	18.9%	902	2.1%	9826	184820.57	4.27

*no limits on attained age. FPC, first primary cancer; SPC, subsequent primary cancer.

Overall, out of 16,531 FPC survivors with SPCs, 10.1% (14,931) of survivors developed one SPC and 1.1% (1,600) had two or more SPCs. Development of an SPC was more common among individuals initially diagnosed with an FPC between the ages of 50 and 64 (44%, 6443 + 765/16,531) and between 65 and 74 (32%, (4778 + 505)/16,531), and least common among those initially diagnosed between 20 and 40, (13%), despite having the longest average exposure of 6.73 years. Among all FPC survivors, 29% (43,334/148,509) were deceased, in those without a SPC, 26% were deceased (32,235/131,978), while the majority (55%, 8197 + 902/16,531) of survivors with a SPC were deceased.

### 3.1 Any site SPC

Among all survivors, 12.1% (17,970/148,509) were diagnosed with one or more SPC at any site (**Table 2**). In comparison to the general Kentucky population, Kentucky cancer survivors had a 21% higher risk of developing a new cancer with a standardized incidence ratio (SIR) of 1.21 (95%CI: 1.19-1.23), and a mean age of 60 at diagnosis of FPC and 71 at SPC. Of the 31 evaluated FPC cancer types, survivors were at increased risk of 26 of them. This effect was consistently observed for men and women with SIR 1.1 (95%CI: 1.04-1.12) and SIR 1.39 (95%CI: 1.36-1.42), respectively, although overall, women were more likely to develop a SPC than men, with an increased risk of almost 40%. Consistent with a prior report,^11^ Kentucky men with a FPC of prostate cancer were the only group less likely to develop a SPC, SIR 0.80 (95% CI: 0.77-0.75). However, when prostate cancer as a FPC was excluded the risk of a SPC in men was SIR 1.42 (95%CI 1.39-1.46), which was similar to risk in women. Of the 30 evaluated FPC cancer types in men, survivors with 20 different FPC were at a risk of SPC. Of the 31 evaluated FPC cancer types in women, survivors with 20 different FPC were at a risk of SPC. Men were diagnosed on average at age 62 for FPC and 72 for SPC, while women had a mean age of diagnosis of FPC at age 58 and 70 for SPC. Among men, the most common FPC and SPC sites numerically were prostate, colon and rectum, and urinary bladder, **Supplementary Figure 1**. In women, breast, colon and rectum, and corpus and uterus were the most frequent FPC sites, while breast, colon and rectum, and lung and bronchus had the highest number of SPCs.

**Table 2:** Risk of developing of SPC from first primary cancer sites in KY adults by sex.

Both Male and Female																			
FPC Sites	Survivors	SPC on Any Site				SPC on Same Site					SPC on Different Sites					Mean Age			
		Observed SPC	SIR	CI Lower	CI Upper	Observed SPC	%	SIR	CI Lower	CI Upper	Observed SPC\	%	SIR	CI Lower	CI Upper	FPC	Any SPC	Same Site SPC	Different Site SPC
All Sites excluding Non-Melanoma Skin	148509	17970	1.21	1.19	1.23	4246	23.6	1.69	1.64	1.75	13724	76.4	1.11	1.09	1.13	59.87	71.13	68.86	71.83
All Sites excluding Non-Melanoma Skin & Prostate	118302	14437	1.40	1.38	1.43	4241	29.4	2.76	2.67	2.84	10196	70.6	1.16	1.14	1.19	58.47	70.06	68.85	70.56
Oral Cavity and Pharynx	3852	741	2.18	2.02	2.34	224	30.2	21.18	18.50	24.14	517	69.8	1.57	1.43	1.71	57.89	68.28	67.18	68.76
Esophagus	408	64	1.70	1.31	2.17	5	7.8	8.95	2.91	20.89	59	92.2	1.59	1.21	2.05	61.01	67.57	66.23	67.68
Stomach	709	80	1.34	1.06	1.66	18	22.5	21.42	12.69	33.85	62	77.5	1.05	0.81	1.35	61.71	71.04	66.28	72.42
Small Intestine	763	77	1.17	0.93	1.47	4	5.2	10.99	2.99	28.14	73	94.8	1.12	0.88	1.41	59.75	71.96	70.78	72.02
Colon and Rectum	15333	1808	1.11	1.06	1.16	260	14.4	1.61	1.42	1.82	1548	85.6	1.05	1.00	1.11	62.64	73.00	71.20	73.30
Anus, Anal Canal and Anorectum	631	96	1.90	1.54	2.33	4	4.2	16.82	4.58	43.07	92	95.8	1.83	1.48	2.25	57.47	67.03	65.90	67.08
Liver, Gallbladder, Intrahepatic Bile Duct and Other Biliary	608	61	1.38	1.05	1.77	5	8.2	4.50	1.46	10.50	56	91.8	1.30	0.98	1.68	60.30	70.83	62.15	71.61
Pancreas	391	35	1.46	1.01	2.03	3	8.6	4.43	0.91	12.94	32	91.4	1.37	0.94	1.93	59.24	65.72	63.89	65.89
Nose, Nasal Cavity and Middle Ear	237	39	1.95	1.39	2.67	5	12.8	159.76	51.87	372.82	34	87.2	1.71	1.18	2.38	59.20	66.35	57.82	67.60
Larynx	1760	401	2.35	2.13	2.59	34	8.5	14.90	10.32	20.83	367	91.5	2.18	1.96	2.42	59.73	68.61	66.56	68.80
Lung and Bronchus	7097	1415	2.41	2.29	2.54	830	58.7	6.58	6.14	7.05	585	41.3	1.27	1.17	1.38	63.32	71.60	71.17	72.21
Bones and Joints	227	28	1.96	1.31	2.84	4	14.3	248.58	67.73	636.45	24	85.7	1.69	1.08	2.51	47.93	58.38	42.21	61.08
Soft Tissue including Heart	774	100	1.60	1.30	1.94	17	17.0	56.37	32.84	90.26	83	83.0	1.33	1.06	1.65	52.51	67.59	65.73	67.97
Melanoma of the Skin	9482	1131	1.31	1.23	1.39	335	29.6	7.64	6.84	8.50	796	70.4	0.97	0.90	1.04	54.84	69.53	67.89	70.22
Breast	29369	3229	1.31	1.26	1.35	1190	36.9	1.92	1.81	2.03	2039	63.1	1.10	1.06	1.15	59.08	69.38	67.40	70.54
Cervix Uteri	1831	155	1.41	1.19	1.64	6	3.9	3.22	1.18	7.00	149	96.1	1.37	1.16	1.61	46.42	60.06	58.29	60.13
Corpus and Uterus, NOS	5838	450	0.91	0.83	1.00	3	0.7	0.11	0.02	0.33	447	99.3	0.96	0.87	1.05	58.47	70.69	56.31	70.79
Ovary	1403	99	1.01	0.82	1.22	1	1.0	0.51	0.01	2.82	98	99.0	1.02	0.82	1.24	55.48	65.60	41.10	65.85
Vulva	538	113	2.97	2.44	3.56	43	38.1	134.79	97.55	181.57	70	61.9	1.85	1.44	2.34	57.32	68.39	71.42	66.53
Prostate	30207	3533	0.77	0.75	0.80	5	0.1	0.01	0.00	0.01	3528	99.9	0.98	0.95	1.01	65.33	75.49	77.62	75.49
Testis	1345	78	1.71	1.35	2.13	13	16.7	16.20	8.62	27.70	65	83.3	1.45	1.12	1.85	35.74	53.36	34.83	57.07
Urinary Bladder	6800	1193	1.52	1.43	1.60	177	14.8	3.08	2.64	3.57	1016	85.2	1.39	1.31	1.48	65.01	73.83	74.01	73.80
Kidney and Renal Pelvis	6138	701	1.26	1.17	1.36	83	11.8	3.92	3.12	4.86	618	88.2	1.16	1.07	1.25	58.72	69.67	63.91	70.44
Eye and Orbit	337	42	1.32	0.95	1.79	6	14.3	92.36	33.89	201.03	36	85.7	1.14	0.80	1.57	59.38	70.72	71.70	70.56
Brain and Other Nervous System	860	56	1.51	1.14	1.97	17	30.4	36.74	21.41	58.83	39	69.6	1.07	0.76	1.46	42.24	54.20	43.96	58.66
Thyroid	5969	433	1.19	1.08	1.31	20	4.6	1.89	1.15	2.92	413	95.4	1.17	1.06	1.29	48.54	64.77	59.94	65.00
Hodgkin Lymphoma	1114	85	1.68	1.34	2.08	0	0.0	0.00	0.00	14.99	85	100.0	1.69	1.35	2.09	39.85	59.80	.	59.80
Non-Hodgkin Lymphoma	6294	847	1.46	1.36	1.56	122	14.4	5.22	4.33	6.23	725	85.6	1.30	1.21	1.40	59.93	70.65	69.93	70.77
Myeloma	1244	95	1.05	0.85	1.29	3	3.2	2.08	0.43	6.09	92	96.8	1.04	0.83	1.27	62.24	70.55	70.47	70.55
Acute Leukemia	409	39	1.59	1.13	2.18	1	2.6	5.56	0.07	30.91	38	97.4	1.57	1.11	2.15	46.63	58.73	62.36	58.63
Chronic Myeloid Leukemia	564	44	1.20	0.87	1.60	0	0.0	0.00	0.00	26.35	44	100.0	1.20	0.87	1.61	52.78	68.24	.	68.24
Male																			
All Sites excluding Non-Melanoma Skin	74001	9958	1.10	1.07	1.12	1794	18.0	1.17	1.12	1.23	8164	82.0	1.08	1.06	1.10	61.55	72.33	69.24	73.01
All Sites excluding Non-Melanoma Skin & Prostate	43794	6425	1.42	1.39	1.46	1789	27.8	3.18	3.04	3.33	4636	72.2	1.17	1.14	1.20	58.95	70.60	69.22	71.13
Oral Cavity and Pharynx	2810	569	2.14	1.97	2.33	165	29.0	17.49	14.92	20.37	404	71.0	1.58	1.43	1.74	57.60	67.91	66.29	68.57
Esophagus	336	50	1.53	1.14	2.02	3	6.0	5.61	1.16	16.39	47	94.0	1.47	1.08	1.95	60.61	67.98	63.98	68.24
Stomach	387	43	1.11	0.80	1.49	6	14.0	9.94	3.65	21.63	37	86.0	0.97	0.68	1.33	61.91	72.81	66.73	73.80
Small Intestine	378	43	1.11	0.81	1.50	4	9.3	19.61	5.34	50.21	39	90.7	1.01	0.72	1.39	59.68	71.21	70.78	71.25
Colon and Rectum	8045	1056	1.06	1.00	1.13	157	14.9	1.65	1.40	1.93	899	85.1	1.00	0.93	1.06	61.89	72.61	69.41	73.17
Anus, Anal Canal and Anorectum	213	33	1.64	1.13	2.31	2	6.1	43.21	5.23	156.07	31	93.9	1.55	1.05	2.20	56.60	67.89	60.04	68.40
Liver, Gallbladder, Intrahepatic Bile Duct and Other Biliary	373	45	1.55	1.13	2.08	3	6.7	3.60	0.74	10.51	42	93.3	1.49	1.07	2.02	59.66	69.26	61.00	69.85
Pancreas	191	14	1.03	0.56	1.72	0	0.0	0.00	0.00	9.81	14	100.0	1.06	0.58	1.77	58.73	62.69	.	62.69
Nose, Nasal Cavity and Middle Ear	149	24	1.71	1.09	2.54	4	16.7	163.29	44.49	418.09	20	83.3	1.42	0.87	2.20	58.82	66.38	60.36	67.58
Larynx	1346	305	2.12	1.89	2.37	25	8.2	11.76	7.61	17.36	280	91.8	1.97	1.75	2.22	60.30	68.96	68.52	69.00
Lung and Bronchus	3393	690	2.04	1.89	2.20	370	53.6	5.06	4.55	5.60	320	46.4	1.21	1.08	1.35	63.62	72.13	71.46	72.90
Bones and Joints	115	14	1.70	0.93	2.85	4	28.6	424.95	115.78	1088.04	10	71.4	1.22	0.58	2.23	47.37	62.23	42.21	70.24
Soft Tissue including Heart	438	70	1.79	1.39	2.26	8	11.4	39.14	16.90	77.13	62	88.6	1.59	1.22	2.04	53.05	68.08	66.90	68.23
Melanoma of the Skin	4936	727	1.33	1.23	1.43	220	30.3	7.19	6.27	8.21	507	69.7	0.98	0.90	1.07	57.56	70.80	69.14	71.52
Breast	141	26	1.55	1.01	2.27	4	15.4	147.00	40.05	376.37	22	84.6	1.31	0.82	1.99	61.26	69.43	61.27	70.91
Prostate	30207	3533	0.77	0.75	0.80	5	0.1	0.01	0.00	0.01	3528	99.9	0.98	0.95	1.01	65.33	75.49	77.62	75.49
Testis	1345	78	1.71	1.35	2.13	13	16.7	16.20	8.62	27.70	65	83.3	1.45	1.12	1.85	35.74	53.36	34.83	57.07
Urinary Bladder	5210	962	1.48	1.39	1.58	148	15.4	2.76	2.33	3.24	814	84.6	1.37	1.27	1.46	65.02	73.78	74.03	73.73
Kidney and Renal Pelvis	3715	454	1.21	1.10	1.33	52	11.5	3.30	2.47	4.33	402	88.5	1.12	1.01	1.24	58.69	69.68	64.42	70.36
Eye and Orbit	178	30	1.51	1.02	2.16	3	10.0	71.85	14.82	209.97	27	90.0	1.37	0.90	1.99	59.30	71.09	77.48	70.38
Brain and Other Nervous System	462	31	1.59	1.08	2.26	9	29.0	32.50	14.86	61.69	22	71.0	1.15	0.72	1.74	41.51	54.71	41.31	60.19
Thyroid	1309	112	1.09	0.90	1.31	2	1.8	1.88	0.23	6.79	110	98.2	1.08	0.89	1.30	51.30	67.28	63.26	67.35
Hodgkin Lymphoma	614	54	1.91	1.44	2.49	0	0.0	0.00	0.00	23.47	54	100.0	1.92	1.44	2.51	40.19	60.08	.	60.08
Non-Hodgkin Lymphoma	3271	465	1.39	1.27	1.53	56	12.0	4.19	3.16	5.44	409	88.0	1.28	1.16	1.41	58.81	70.36	68.15	70.66
Myeloma	692	62	1.13	0.86	1.44	1	1.6	1.10	0.03	6.15	61	98.4	1.13	0.86	1.45	61.55	71.44	64.08	71.56
Acute Leukemia	227	23	1.54	0.97	2.31	1	4.3	9.09	0.12	50.58	22	95.7	1.49	0.93	2.25	46.10	54.26	62.36	53.90
Chronic Myeloid Leukemia	290	21	1.08	0.67	1.65	0	0.0	0.00	0.00	46.87	21	100.0	1.08	0.67	1.66	51.51	69.40	.	69.40
Female																			
All Sites excluding Non-Melanoma Skin	74508	8012	1.39	1.36	1.42	2452	30.6	2.51	2.41	2.61	5560	69.4	1.16	1.13	1.19	58.19	69.62	68.58	70.08
All Sites excluding Non-Melanoma Skin & Prostate	74508	8012	1.39	1.36	1.42	2452	30.6	2.51	2.41	2.61	5560	69.4	1.16	1.13	1.19	58.19	69.62	68.58	70.08
Oral Cavity and Pharynx	1042	172	2.29	1.96	2.66	59	34.3	51.68	39.34	66.67	113	65.7	1.53	1.26	1.84	58.67	69.52	69.67	69.44
Esophagus	72	14	2.79	1.53	4.69	2	14.3	84.39	10.22	304.85	12	85.7	2.40	1.24	4.20	62.87	66.09	69.62	65.50
Stomach	322	37	1.77	1.24	2.43	12	32.4	50.70	26.20	88.56	25	67.6	1.21	0.78	1.78	61.46	68.98	66.06	70.38
Small Intestine	385	34	1.26	0.87	1.77	0	0.0	0.00	0.00	23.06	34	100.0	1.27	0.88	1.78	59.82	72.92	.	72.92
Colon and Rectum	7288	752	1.19	1.10	1.28	103	13.7	1.56	1.27	1.89	649	86.3	1.14	1.06	1.24	63.47	73.55	73.94	73.49
Anus, Anal Canal and Anorectum	418	63	2.08	1.60	2.66	2	3.2	10.44	1.26	37.73	61	96.8	2.02	1.55	2.60	57.92	66.58	71.75	66.41
Liver, Gallbladder, Intrahepatic Bile Duct and Other Biliary	235	16	1.04	0.60	1.70	2	12.5	7.23	0.88	26.10	14	87.5	0.93	0.51	1.56	61.30	75.25	63.88	76.87
Pancreas	200	21	2.02	1.25	3.09	3	14.3	9.95	2.05	29.09	18	85.7	1.78	1.06	2.82	59.72	67.74	63.89	68.38
Nose, Nasal Cavity and Middle Ear	88	15	2.55	1.42	4.20	1	6.7	147.03	3.72	819.23	14	93.3	2.38	1.30	4.00	59.84	66.31	47.66	67.64
Larynx	414	96	3.62	2.93	4.42	9	9.4	57.90	26.48	109.92	87	90.6	3.30	2.65	4.07	57.85	67.49	61.15	68.15
Lung and Bronchus	3704	725	2.91	2.70	3.13	460	63.4	8.70	7.92	9.53	265	36.6	1.35	1.19	1.52	63.05	71.10	70.93	71.40
Bones and Joints	112	14	2.33	1.27	3.91	0	0.0	0.00	0.00	552.33	14	100.0	2.33	1.27	3.92	48.50	54.53	.	54.53
Soft Tissue including Heart	336	30	1.27	0.86	1.82	9	30.0	92.62	42.35	175.83	21	70.0	0.90	0.55	1.37	51.80	66.45	64.69	67.20
Melanoma of the Skin	4546	404	1.28	1.16	1.41	115	28.5	8.66	7.15	10.40	289	71.5	0.95	0.85	1.07	51.89	67.26	65.51	67.96
Breast	29228	3203	1.31	1.26	1.35	1186	37.0	1.91	1.80	2.02	2017	63.0	1.10	1.05	1.15	59.06	69.37	67.42	70.52
Cervix Uteri	1831	155	1.41	1.19	1.64	6	3.9	3.22	1.18	7.00	149	96.1	1.37	1.16	1.61	46.42	60.06	58.29	60.13
Corpus and Uterus, NOS	5838	450	0.91	0.83	1.00	3	0.7	0.11	0.02	0.33	447	99.3	0.96	0.87	1.05	58.47	70.69	56.31	70.79
Ovary	1403	99	1.01	0.82	1.22	1	1.0	0.51	0.01	2.82	98	99.0	1.02	0.82	1.24	55.48	65.60	41.10	65.85
Vulva	538	113	2.97	2.44	3.56	43	38.1	134.79	97.55	181.57	70	61.9	1.85	1.44	2.34	57.32	68.39	71.42	66.53
Urinary Bladder	1590	231	1.68	1.47	1.91	29	12.6	7.46	4.99	10.71	202	87.4	1.51	1.31	1.74	64.99	74.04	73.91	74.06
Kidney and Renal Pelvis	2423	247	1.37	1.21	1.55	31	12.6	5.71	3.88	8.11	216	87.4	1.24	1.08	1.41	58.78	69.65	63.05	70.60
Eye and Orbit	159	12	1.01	0.52	1.76	3	25.0	129.26	26.66	377.77	9	75.0	0.76	0.34	1.43	59.46	69.78	65.93	71.06
Brain and Other Nervous System	398	25	1.43	0.92	2.11	8	32.0	43.08	18.60	84.89	17	68.0	0.98	0.57	1.57	43.08	53.57	46.93	56.69
Thyroid	4660	321	1.23	1.10	1.37	18	5.6	1.89	1.12	2.99	303	94.4	1.20	1.07	1.35	47.77	63.89	59.57	64.15
Hodgkin Lymphoma	500	31	1.39	0.94	1.97	0	0.0	0.00	0.00	41.49	31	100.0	1.39	0.94	1.97	39.42	59.30	.	59.30
Non-Hodgkin Lymphoma	3023	382	1.55	1.40	1.72	66	17.3	6.59	5.10	8.39	316	82.7	1.34	1.20	1.49	61.13	70.99	71.44	70.90
Myeloma	552	33	0.94	0.65	1.32	2	6.1	3.75	0.45	13.53	31	93.9	0.89	0.61	1.27	63.11	68.88	73.67	68.57
Acute Leukemia	182	16	1.68	0.96	2.72	0	0.0	0.00	.	.	16	100.0	1.69	0.97	2.75	47.29	65.15	.	65.15
Chronic Myeloid Leukemia	274	23	1.32	0.84	1.99	0	0.0	0.00	0.00	60.19	23	100.0	1.33	0.84	1.99	54.12	67.19	.	67.19

*Includes Sites with Survivors 200+ for both Gender Combined. FPC, first primary cancer; SPC, subsequent primary cancer; CI, confidence interval; SIR, standardized incidence ratio.

Interestingly, while numerically the most SPCs (3533) occurred in men with an FPC of prostate cancer, their risk of a SPC was lower than the Kentucky general population with an SIR 0.77 (95% CI:0.75-0.80), **Table 2**. Men with a FPC of the oral cavity, SIR 2.14 (95% CI: 1.97-2.33), larynx, SIR 2.12 (95% CI:1.89-2.37), and lung, SIR 2.04 (95% CI:1.89-2.20) had the highest risk of a SPC, while women with a FPC of the larynx, SIR 3.62 (95% CI:2.93-4.42), vulva, SIR 2.97 (95% CI:2.44-3.56), and lung and bronchus cancers, SIR 2.91 (95% CI:2.70-3.13) were at the highest risk of SPC. Unlike men with an FPC of prostate cancer, women surviving an FPC were not at a decreased risk of any SPC.

### 3.2 Same site SPC

Among all survivors with an FPC, 2.9% (4246/148,509) were diagnosed with an SPC at the same site, representing 23.6% (4246/17970) of all SPCs, **Table 2**. FPC patients had an overall higher risk of cancer than the general population, SIR 1.69 (95% CI:1.64-1.75) with average diagnostic age at 69. For same site SPCs with more than 40 cases, the most common same site SPCs were breast (1190. 36.9% of total SPCs), lung (830, 58.7%), melanoma (335, 29.6%), and oral cavity (224, 30.2%), while individuals with a FPC of the vulva, SIR 135 (95% CI:97.55-181.57), oral cavity, SIR 21.2 (95% CI:18.50-24.14), and lung, SIR 6.58 (95% CI:6.14-7.05) had the highest risk of a same site SPC.

Of the 30 evaluated FPC cancer types in men, survivors with 22 different FPC were at significantly higher risk of a same site SPC than the general Kentucky population. A total of 1794 (18.0%) same site SPCs were observed for Kentucky male FPC patients. The most commonly observed same site SPCs among men were lung (370,53.6%), melanoma (220,30.3%) and oral cavity and pharynx (165,29.0%). Of the 31 evaluated FPC cancer types in women, survivors with 23 different FPC were at significantly higher risk of SPC than the general Kentucky population. 2452 (30.6%) of same site SPCs occurred for female population during study period. The most commonly observed same site SPCs among women were breast (1186,37.0%), lung (460,63.4%) and colon (103, 13.7%). Excluding gender specific cancers, lung and oral cavity and pharynx cancers were among the most common same site SPCs in both men and women. Interestingly, risk of same site SPC in the oral cavity, SIR 17.5 (95% CI: 14.9-20.4) for men versus SIR 51.7 (95% CI: 39.3-66.7) for women and lung cancer SIR 5.06 (95% CI: 4.55-5.60) for men versus SIR 8.7 (95% CI: 7.92-9.53) was significantly higher in female cancer survivors when compared to males.

### 3.3 Different site SPC

Among all survivors with an FPC, 9.2% (13,724/148,509) were diagnosed with an SPC at a different site and had an overall higher risk of cancer than the general population, SIR 1.11 (95% CI:1.09-1.13), **Table 2**. With the exception of lung cancer, survivors of an FPC were more likely to have a different site SPC. Among the 7097 individuals with lung cancer, 1415 developed a SPC, 830 (58.7%) in the same site and 585 (41.3%) at a different site. SPCs were most commonly observed in those with an FPC of prostate (3528,99%+), breast (2039,63.1%) and colon (1548,85.6%), while individuals with an FPC of the larynx, SIR 2.18 (95% CI:1.96-2.42), vulva, SIR 1.85 (95% CI:1.44-2.34), and anus, SIR 1.83 (95% CI:1.48-2.25) had the highest risk of a different site SPC. Among the 31 cancer sites evaluated, survivors with 19 different FPC were at risk of a SPC, while the remaining 11 were at the same risk as the general population.

Of the 30 evaluated FPC cancer types in men, survivors with 14 different FPC were at significantly higher risk of a different site SPC than the general Kentucky population. The most common FPCs among men with a different site SPC were prostate (3528, 99%+), colon (899,85.1%) and urinary bladder (165,84.6%), while men with a FPC of the larynx, SIR 1.97 (95% CI:1.75-2.22), Hodgkin lymphoma, SIR 1.92 (95% CI:1.44-2.51), and oral cavity and pharynx, SIR 1.58 (95% CI:1.43-1.74) had the highest risk of a different site SPC. Of the 31 evaluated FPC cancer types in women, survivors with 16 different FPC were at significantly higher risk of a different site SPC than the general Kentucky population. The most commonly FPCs among women with a different site SPC were breast (2017,63.0%), colon (649,86.3%) and uterine (447,99.3%), while women with an FPC of the larynx, SIR 3.30 (95% CI:2.65-4.07), anus, SIR 2.02 (95% CI:1.55-2.60), and vulva, SIR 1.85 (95% CI:1.44-1.34) had the highest risk of a different site SPC.

### 3.4 Appalachian

Increased risk for any site SPC among cancer survivors was consistently observed for non-Appalachian and Appalachian residents with SIR 1.19 (95%CI: 1.17-1.21) and SIR 1.26 (95%CI: 1.23-1.30), respectively, with Appalachian residents at significantly higher risk than non-Appalachian residents. (**Table 3**). Non-Appalachian residents were diagnosed on average at age 59.9 for FPC and 71.3 for SPC and Appalachian residents had a mean age of diagnosis of FPC at age 59.7 and 70.6 for SPC. The highest risk for any SPC in non-Appalachian survivors was seen with FPC of the vulva, SIR 2.74 (95% CI:2.15-3.43), lung and bronchus, SIR 2.44 (95% CI:2.29-2.59), and bones and joints, SIR 2.30 (95% CI:1.46-3.45). Appalachian survivors were at highest risk of SPC when the FPC was vulva, SIR 3.55 (95% CI:2.51-4.87), acute leukemia, SIR 2.87 (95% CI:1.57-4.81), and larynx, SIR 2.72 (95% CI:2.30-3.18). The most frequently observed FPC and SPC sites in both non-Appalachian and Appalachian residents were prostate, breast, and colon and rectum, **Supplementary Figure 1**.

**Table 3:** Risk of developing SPC from first primary cancer sites in KY adults by Appalachia status.

Non-Appalachia																			
FPC Sites	Survivors	SPC on Any Site				SPC on Same Site					SPC on Different Sites					Mean Age			
		Observed SPC	SIR	CI Lower	CI Upper	Observed SPC	%	SIR	CI Lower	CI Upper	Observed SPC	%	SIR	CI Lower	CI Upper	FPC	Any SPC	Same Site SPC	Different Site SPC
All Sites excluding Non-Melanoma Skin	107121	12833	1.19	1.17	1.21	3051	23.8%	1.66	1.60	1.72	9782	76.2%	1.09	1.07	1.11	59.93	71.33	69.11	72.03
All Sites excluding Non-Melanoma Skin & Prostate	84734	10261	1.39	1.36	1.41	3047	29.7%	2.73	2.64	2.83	7214	70.3%	1.15	1.12	1.18	58.55	70.29	69.09	70.79
Oral Cavity and Pharynx	2746	538	2.21	2.02	2.40	169	31.4%	22.43	19.18	26.08	369	68.6%	1.56	1.41	1.73	57.91	68.59	67.45	69.11
Esophagus	304	45	1.64	1.19	2.19	3	6.7%	7.41	1.53	21.65	42	93.3%	1.55	1.12	2.10	60.32	66.65	66.53	66.66
Stomach	469	52	1.37	1.02	1.79	13	25.0%	23.59	12.56	40.34	39	75.0%	1.04	0.74	1.42	62.18	71.55	69.02	72.39
Small Intestine	556	57	1.16	0.88	1.51	3	5.3%	10.86	2.24	31.74	54	94.7%	1.11	0.83	1.45	60.18	71.19	67.56	71.39
Colon and Rectum	10775	1248	1.09	1.03	1.15	183	14.7%	1.60	1.38	1.85	1065	85.3%	1.03	0.97	1.09	62.81	73.29	71.68	73.57
Anus, Anal Canal and Anorectum	487	83	2.10	1.67	2.60	4	4.8%	21.53	5.87	55.14	79	95.2%	2.01	1.59	2.50	57.88	66.19	65.90	66.20
Liver, Gallbladder, Intrahepatic Bile Duct and Other Biliary	456	46	1.38	1.01	1.85	5	10.9%	5.82	1.89	13.57	41	89.1%	1.27	0.91	1.72	60.13	70.11	62.15	71.08
Pancreas	291	26	1.44	0.94	2.11	3	11.5%	5.87	1.21	17.16	23	88.5%	1.31	0.83	1.97	58.75	67.76	63.89	68.26
Nose, Nasal Cavity and Middle Ear	171	24	1.69	1.08	2.51	4	16.7%	179.40	48.88	459.33	20	83.3%	1.41	0.86	2.18	59.23	65.62	60.36	66.67
Larynx	1126	248	2.17	1.91	2.46	20	8.1%	13.15	8.03	20.30	228	91.9%	2.02	1.77	2.30	60.24	69.38	66.54	69.63
Lung and Bronchus	5005	1029	2.44	2.29	2.59	588	57.1%	6.48	5.96	7.02	441	42.9%	1.33	1.21	1.46	63.67	72.02	71.73	72.41
Bones and Joints	159	23	2.30	1.46	3.45	4	17.4%	358.56	97.70	918.06	19	82.6%	1.90	1.14	2.97	47.55	57.45	42.21	60.66
Soft Tissue including Heart	551	73	1.61	1.26	2.03	10	13.7%	45.19	21.67	83.11	63	86.3%	1.40	1.08	1.79	52.32	67.54	69.50	67.23
Melanoma of the Skin	6861	801	1.28	1.19	1.37	235	29.3%	7.39	6.48	8.40	566	70.7%	0.95	0.88	1.04	54.97	69.59	67.85	70.31
Breast	21794	2353	1.28	1.23	1.33	880	37.4%	1.91	1.78	2.04	1473	62.6%	1.07	1.02	1.13	59.09	69.61	67.53	70.85
Cervix Uteri	1269	105	1.39	1.14	1.68	4	3.8%	3.08	0.84	7.89	101	96.2%	1.36	1.11	1.65	46.17	60.43	57.68	60.54
Corpus and Uterus, NOS	3907	311	0.94	0.84	1.05	3	1.0%	0.17	0.03	0.49	308	99.0%	0.98	0.87	1.10	58.70	70.72	56.31	70.86
Ovary	984	70	1.02	0.79	1.29	0	0.0%	0.00	0.00	2.67	70	100.0%	1.04	0.81	1.31	55.57	65.80	.	65.80
Vulva	377	75	2.74	2.15	3.43	28	37.3%	123.78	82.25	178.89	47	62.7%	1.73	1.27	2.30	57.52	68.64	71.94	66.67
Prostate	22387	2572	0.76	0.73	0.79	4	0.2%	0.01	0.00	0.01	2568	99.8%	0.96	0.92	1.00	65.13	75.51	79.04	75.50
Testis	1004	56	1.58	1.20	2.06	9	16.1%	15.21	6.96	28.88	47	83.9%	1.35	0.99	1.80	36.00	53.18	34.09	56.84
Urinary Bladder	4809	836	1.49	1.39	1.59	130	15.6%	3.16	2.64	3.75	706	84.4%	1.36	1.26	1.46	65.26	74.24	73.96	74.29
Kidney and Renal Pelvis	4454	533	1.31	1.20	1.43	68	12.8%	4.38	3.40	5.55	465	87.2%	1.19	1.09	1.30	58.75	70.10	65.36	70.79
Eye and Orbit	229	27	1.39	0.92	2.03	6	22.2%	150.38	55.19	327.31	21	77.8%	1.09	0.67	1.66	58.34	70.54	71.70	70.21
Brain and Other Nervous System	631	47	1.71	1.26	2.28	15	31.9%	43.90	24.57	72.41	32	68.1%	1.18	0.81	1.67	41.77	54.23	42.82	59.58
Thyroid	4041	283	1.16	1.03	1.30	9	3.2%	1.26	0.58	2.39	274	96.8%	1.15	1.02	1.30	48.36	65.04	61.52	65.16
Hodgkin Lymphoma	825	59	1.55	1.18	2.00	0	0.0%	0.00	0.00	19.96	59	100.0%	1.56	1.18	2.01	39.86	59.85	.	59.85
Non-Hodgkin Lymphoma	4581	604	1.44	1.33	1.56	87	14.4%	5.15	4.12	6.35	517	85.6%	1.28	1.18	1.40	59.86	70.80	69.90	70.95
Myeloma	922	67	0.99	0.77	1.26	2	3.0%	1.81	0.22	6.53	65	97.0%	0.98	0.76	1.25	62.51	71.54	73.67	71.47
Acute Leukemia	316	25	1.28	0.83	1.89	0	0.0%	0.00			25	100.0%	1.29	0.83	1.90	47.20	58.42		58.42
Chronic Myeloid Leukemia	387	30	1.24	0.83	1.76	0	0.0%	0.00	0.00	39.81	30	100.0%	1.24	0.84	1.77	52.58	69.81	.	69.81
Appalachia																			
FPC Sites	Survivors	SPC on Any Site				SPC on Same Site					SPC on Different Sites					Mean Age			
		Observed SPC	SIR	CI Lower	CI Upper	Observed SPC	%	SIR	CI Lower	CI Upper	Observed SPC	%	SIR	CI Lower	CI Upper	FPC	Any SPC	Same Site SPC	Different Site SPC
All Sites excluding Non-Melanoma Skin	41388	5137	1.26	1.23	1.30	1195	23.3%	1.80	1.70	1.91	3942	76.7%	1.16	1.12	1.20	59.70	70.60	68.22	71.32
All Sites excluding Non-Melanoma Skin & Prostate	33568	4176	1.44	1.40	1.48	1194	28.6%	2.81	2.66	2.98	2982	71.4%	1.20	1.16	1.25	58.26	69.49	68.22	69.99
Oral Cavity and Pharynx	1106	203	2.10	1.82	2.41	55	27.1%	18.07	13.62	23.53	148	72.9%	1.58	1.34	1.86	57.84	67.48	66.36	67.90
Esophagus	104	19	1.88	1.13	2.93	2	10.5%	13.01	1.58	46.99	17	89.5%	1.71	0.99	2.73	63.00	69.73	65.78	70.19
Stomach	240	28	1.29	0.86	1.86	5	17.9%	17.27	5.61	40.30	23	82.1%	1.07	0.68	1.61	60.79	70.08	59.17	72.45
Small Intestine	207	20	1.20	0.74	1.86	1	5.0%	11.40	0.29	63.50	19	95.0%	1.15	0.69	1.80	58.61	74.17	80.42	73.84
Colon and Rectum	4558	560	1.16	1.07	1.26	77	13.8%	1.64	1.30	2.05	483	86.3%	1.11	1.01	1.22	62.23	72.36	70.08	72.72
Anus, Anal Canal and Anorectum	144	13	1.20	0.64	2.04	0	0.0%	0.00	0.00	70.89	13	100.0%	1.20	0.64	2.05	56.09	72.38	.	72.38
Liver, Gallbladder, Intrahepatic Bile Duct and Other Biliary	152	15	1.35	0.76	2.23	0	0.0%	0.00	0.00	14.67	15	100.0%	1.39	0.77	2.28	60.80	73.05	.	73.05
Pancreas	100	9	1.51	0.69	2.86	0	0.0%	0.00	0.00	22.14	9	100.0%	1.55	0.71	2.94	60.65	59.82	.	59.82
Nose, Nasal Cavity and Middle Ear	66	15	2.61	1.46	4.31	1	6.7%	111.11	2.81	619.05	14	93.3%	2.44	1.33	4.10	59.12	67.52	47.66	68.94
Larynx	634	153	2.72	2.30	3.18	14	9.2%	18.43	10.07	30.91	139	90.8%	2.50	2.10	2.95	58.81	67.35	66.60	67.43
Lung and Bronchus	2092	386	2.34	2.12	2.59	242	62.7%	6.86	6.02	7.78	144	37.3%	1.11	0.94	1.31	62.50	70.49	69.81	71.63
Bones and Joints	68	5	1.18	0.38	2.74	0	0.0%	0.00	0.00	747.35	5	100.0%	1.18	0.38	2.75	48.81	62.67	.	62.67
Soft Tissue including Heart	223	27	1.55	1.02	2.25	7	25.9%	87.21	35.06	179.68	20	74.1%	1.15	0.70	1.78	52.96	67.73	60.35	70.31
Melanoma of the Skin	2621	330	1.38	1.24	1.54	100	30.3%	8.27	6.73	10.06	230	69.7%	1.01	0.89	1.15	54.50	69.39	68.00	69.99
Breast	7575	876	1.38	1.29	1.48	310	35.4%	1.94	1.73	2.17	566	64.6%	1.19	1.10	1.30	59.03	68.74	67.04	69.67
Cervix Uteri	562	50	1.44	1.07	1.90	2	4.0%	3.52	0.43	12.72	48	96.0%	1.41	1.04	1.87	46.97	59.28	59.50	59.27
Corpus and Uterus, NOS	1931	139	0.87	0.73	1.02	0	0.0%	0.00	0.00	0.42	139	100.0%	0.92	0.77	1.08	58.00	70.62	.	70.62
Ovary	419	29	0.98	0.65	1.40	1	3.4%	1.67	0.04	9.33	28	96.6%	0.96	0.64	1.39	55.26	65.09	41.10	65.95
Vulva	161	38	3.55	2.51	4.87	15	39.5%	161.64	90.47	266.61	23	60.5%	2.17	1.37	3.25	56.84	67.90	70.44	66.24
Prostate	7820	961	0.83	0.78	0.88	1	0.1%	0.00	0.00	0.02	960	99.9%	1.04	0.97	1.11	65.88	75.45	71.92	75.45
Testis	341	22	2.14	1.34	3.23	4	18.2%	18.97	5.17	48.56	18	81.8%	1.78	1.06	2.82	34.97	53.82	36.50	57.67
Urinary Bladder	1991	357	1.58	1.42	1.75	47	13.2%	2.88	2.11	3.83	310	86.8%	1.48	1.32	1.66	64.41	72.86	74.13	72.67
Kidney and Renal Pelvis	1684	168	1.13	0.96	1.31	15	8.9%	2.66	1.49	4.39	153	91.1%	1.07	0.91	1.25	58.64	68.30	57.32	69.38
Eye and Orbit	108	15	1.22	0.68	2.00	0	0.0%	0.00	0.00	147.18	15	100.0%	1.22	0.68	2.01	61.57	71.03	.	71.03
Brain and Other Nervous System	229	9	0.94	0.43	1.78	2	22.2%	16.53	2.00	59.72	7	77.8%	0.74	0.30	1.53	43.54	54.08	52.50	54.53
Thyroid	1928	150	1.26	1.06	1.48	11	7.3%	3.20	1.60	5.73	139	92.7%	1.20	1.01	1.42	48.94	64.26	58.65	64.70
Hodgkin Lymphoma	289	26	2.08	1.36	3.04	0	0.0%	0.00	0.00	60.17	26	100.0%	2.09	1.36	3.06	39.80	59.67	.	59.67
Non-Hodgkin Lymphoma	1713	243	1.51	1.33	1.72	35	14.4%	5.40	3.76	7.51	208	85.6%	1.35	1.17	1.55	60.09	70.26	70.01	70.30
Myeloma	322	28	1.23	0.81	1.77	1	3.6%	3.00	0.08	16.69	27	96.4%	1.20	0.79	1.75	61.48	68.20	64.08	68.35
Acute Leukemia	93	14	2.87	1.57	4.81	1	7.1%	33.33	0.44	185.46	13	92.9%	2.68	1.43	4.58	44.67	59.27	62.36	59.03
Chronic Myeloid Leukemia	177	14	1.12	0.61	1.87	0	0.0%	0.00	0.00	77.91	14	100.0%	1.12	0.61	1.88	53.22	64.90	.	64.90

Rates of same site SPC between Appalachia and non-Appalachia were similar, with 23% of all SPC occurring in the same site of FPC, **Table 3**. Among cases when the SPC count was 15 or more, the most commonly observed same site SPCs among non-Appalachian residents were breast (880), lung (588) and colon (183), while individuals with a FPC of the vulva, SIR 124 (95% CI:82-179), oral cavity, SIR 22.4 (95% CI:19.2-26.1), and CNS, SIR 43.9 (95% CI:24.5-72.4) had the highest risk of a same site SPC. Among individuals residing in Appalachia, the most commonly observed same site SPCs among Appalachian residents were breast (310), lung (242) and melanoma (100), while individuals with an FPC of the vulva, SIR 161.6 (95% CI:90-266), oral cavity, SIR 18.1 (95% CI:13.6-23.5) and melanoma, SIR 8.27 (95% CI:6.73-7.78) had the highest risk of a same site SPC.

The majority of SPCs occurred in different sites (~76%) for Appalachian and non-Appalachian populations. Increased risk for different site SPC among cancer survivors was consistently observed for non-Appalachian and Appalachian residents with SIR 1.09 (95%CI: 1.07-1.11) and SIR 1.16 (95%CI: 1.12-1.20), respectively, with Appalachian residents at significantly higher risk than non-Appalachian residents. The most commonly observed different site SPCs among non-Appalachian residents occurred in those with an FPC of prostate (2568), breast (1473), and colon (1065), while individuals with an FPC of the larynx, SIR 2.02 (95% CI:1.77-2.03), anus, SIR 2.01 (95% CI:1.59-2.02), and vulva, SIR 1.73 (95% CI:1.27-2.30) had the highest risk of a different site SPC. Among individuals residing in Appalachia, the most commonly observed different site SPCs among Appalachian residents occurred in those with a FPC of prostate (960), breast (566), and colon (483), while individuals with a FPC of the larynx, SIR 2.50 (95% CI:2.10-2.95), oral cavity and pharynx, SIR 1.58 (95% CI:1.34-1.86), and urinary bladder, SIR 1.48 (95% CI:1.32-1.66) had the highest risk of a different site SPC.

### 3.5 Risk by FPC

**Figure 1:**
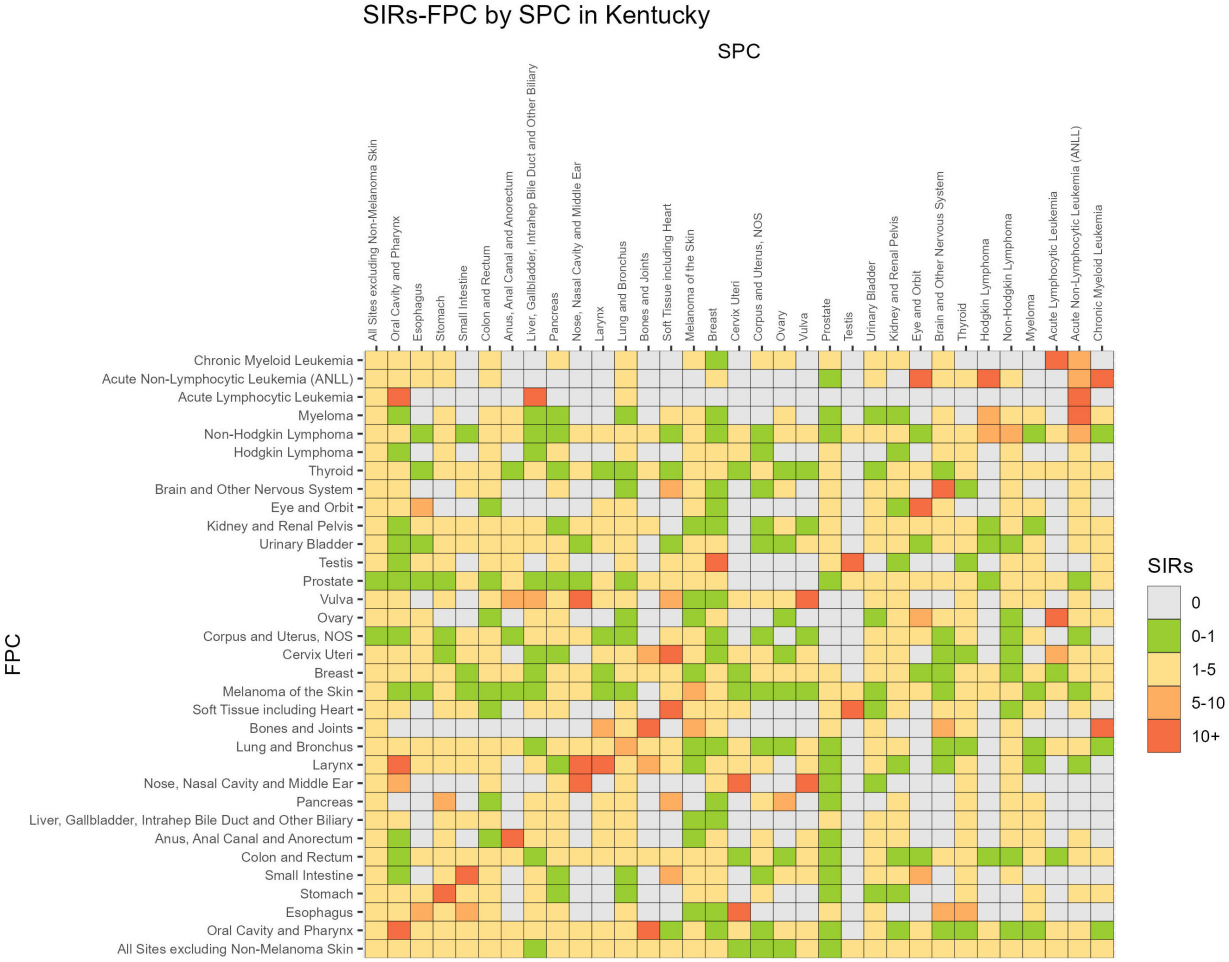
SIR, Standardized Incidence Ratio; FPC, First primary cancer; SPC, Subsequent primary cancer.

Associations between FPC and SPC for individual cancer sites are displayed in **Figure 1** (24). The SIRs were compressed if values were larger than 10 and the diagonal values indicate SIRs for same site SPCs. The highest risk of SPC appeared to be a same site, with 17/31 FPC cancers having an SIR of 5 or greater for a same site SPC, however all cancers had an SIR from 1-5 for increased risk of a different site SPC, and survivors with a FPC of esophageal cancer had SIR > 5 for a SPC small intestine, cervical, eye and thyroids cancers. Similar associations were observed for men and women and Appalachian versus non-Appalachian residents and are presented in **Supplementary Figures 2** and **3**.

### 3.6 Risk factor associated cancers

We next evaluated 12 smoking related FPCs (**Table 4**), which accounted for 37.1% (6663/17,970) of all SPCs. Overall, survivors with a smoking associated FPC had significantly higher risks of any site, SIR 1.53 (95% CI:1.50-1.57), same site, SIR 1.93 (95% CI:1.88-1.99) and different site SPC, SIR 1.10 (95% CI:2.1.05-1.14) when compared to the general population which was consistent across men, women, Appalachian and non-Appalachian residents. The majority 65.7% (4375/6663) of SPCs were in another smoking related site, with women having a higher risk compared to men, SIR 2.38 (95% CI:2.26-2.50) and SIR 1.75 (95% CI:1.69-1.82), respectively. Interestingly, residing in Appalachian was not associated with increased risk of a smoking associated SPC when compared to non-Appalachian residents.

**Table 4:** SPC for smoke-related cancer sites by sex and Appalachia status.

Genders	FPC Sites	Observed SPC	SPC on Any Site				SPC on Risk Factor Related Sites					SPC on non-Risk Factor Related Sites				
			Expected SPC	SIR	CI Lower	CI Upper	Observed SPC	%	SIR	CI Lower	CI Upper	Observed SPC	%	SIR	CI Lower	CI Upper
Male and female	**Total Combined 12 Sites**	**6663**	**4347.75**	**1.53**	**1.50**	**1.57**	**4375**	**65.7%**	**1.93**	**1.88**	**1.99**	**2288**	**34.3%**	**1.10**	**1.05**	**1.14**
	Oral Cavity and Pharynx	741	340.63	2.18	2.02	2.34	570	76.9%	3.18	2.92	3.45	171	23.1%	1.06	0.91	1.23
	Esophagus	64	37.61	1.70	1.31	2.17	46	71.9%	2.26	1.66	3.02	18	28.1%	1.04	0.62	1.65
	Stomach	80	59.84	1.34	1.06	1.66	51	63.8%	1.64	1.22	2.16	29	36.3%	1.01	0.67	1.44
	Colon and Rectum	1808	1630.15	1.11	1.06	1.16	997	55.1%	1.18	1.11	1.26	811	44.9%	1.03	0.96	1.11
	Liver	37	25.5	1.45	1.02	2.00	25	67.6%	1.90	1.23	2.81	12	32.4%	0.97	0.50	1.70
	Pancreas	35	24.04	1.46	1.01	2.02	20	57.1%	1.65	1.01	2.56	15	42.9%	1.26	0.70	2.07
	Larynx	401	170.56	2.35	2.13	2.59	314	78.3%	3.44	3.07	3.84	87	21.7%	1.10	0.88	1.35
	Lung and Bronchus	1415	586.76	2.41	2.29	2.54	1118	79.0%	3.71	3.49	3.93	297	21.0%	1.04	0.93	1.17
	Cervix Uteri	155	110.29	1.41	1.19	1.64	82	52.9%	1.86	1.48	2.31	73	47.1%	1.10	0.86	1.39
	Kidney and Renal Pelvis	701	554.74	1.26	1.17	1.36	381	54.4%	1.33	1.20	1.47	320	45.6%	1.20	1.07	1.33
	Urinary Bladder	1193	787.33	1.52	1.43	1.60	754	63.2%	1.77	1.64	1.90	439	36.8%	1.22	1.11	1.34
	Acute Non-Lymphocytic Leukemia (ANLL)	30	19.63	1.53	1.03	2.18	15	50.0%	1.55	0.87	2.56	15	50.0%	1.50	0.84	2.48
Male	**Total Combined 12 Sites**	**4196**	**2883.83**	**1.46**	**1.41**	**1.50**	**2808**	**66.9%**	**1.75**	**1.69**	**1.82**	**1388**	**33.1%**	**1.08**	**1.03**	**1.14**
	Oral Cavity and Pharynx	569	265.6	2.14	1.97	2.33	431	75.7%	2.95	2.68	3.24	138	24.3%	1.16	0.97	1.36
	Esophagus	50	32.6	1.53	1.14	2.02	34	68.0%	1.89	1.31	2.64	16	32.0%	1.10	0.63	1.78
	Stomach	43	38.89	1.11	0.80	1.49	29	67.4%	1.35	0.90	1.94	14	32.6%	0.81	0.44	1.35
	Colon and Rectum	1056	996.95	1.06	1.00	1.13	631	59.8%	1.14	1.05	1.23	425	40.2%	0.96	0.87	1.06
	Liver	32	17.98	1.78	1.22	2.51	24	75.0%	2.45	1.57	3.65	8	25.0%	0.98	0.42	1.92
	Pancreas	14	13.63	1.03	0.56	1.72	7	50.0%	0.93	0.37	1.92	7	50.0%	1.14	0.46	2.36
	Larynx	305	144.06	2.12	1.89	2.37	238	78.0%	2.99	2.62	3.39	67	22.0%	1.04	0.81	1.32
	Lung and Bronchus	690	337.76	2.04	1.89	2.20	542	78.6%	2.88	2.65	3.14	148	21.4%	0.99	0.83	1.16
	Cervix Uteri	0	0	.	.	.	0		.	.	.	0		.	.	.
	Kidney and Renal Pelvis	454	374.58	1.21	1.10	1.33	259	57.0%	1.25	1.10	1.41	195	43.0%	1.16	1.01	1.34
	Urinary Bladder	962	649.81	1.48	1.39	1.58	603	62.7%	1.66	1.53	1.80	359	37.3%	1.25	1.13	1.39
	Acute Non-Lymphocytic Leukemia (ANLL)	18	11.73	1.53	0.91	2.43	8	44.4%	1.26	0.54	2.47	10	55.6%	1.87	0.89	3.43
Female	**Total Combined 12 Sites**	**2467**	**1463.91**	**1.69**	**1.62**	**1.75**	**1567**	**63.5%**	**2.38**	**2.26**	**2.50**	**900**	**36.5%**	**1.12**	**1.05**	**1.19**
	Oral Cavity and Pharynx	172	75.02	2.29	1.96	2.66	139	80.8%	4.20	3.53	4.95	33	19.2%	0.79	0.54	1.11
	Esophagus	14	5.01	2.79	1.53	4.69	12	85.7%	5.17	2.67	9.04	2	14.3%	0.74	0.08	2.68
	Stomach	37	20.95	1.77	1.24	2.43	22	59.5%	2.31	1.45	3.50	15	40.5%	1.31	0.73	2.17
	Colon and Rectum	752	633.2	1.19	1.10	1.28	366	48.7%	1.26	1.13	1.40	386	51.3%	1.13	1.02	1.24
	Liver	5	7.52	0.66	0.21	1.55	1	20.0%	0.30	0.00	1.66	4	80.0%	0.96	0.26	2.46
	Pancreas	21	10.4	2.02	1.25	3.09	13	61.9%	2.83	1.51	4.84	8	38.1%	1.38	0.59	2.71
	Larynx	96	26.5	3.62	2.93	4.42	76	79.2%	6.55	5.16	8.20	20	20.8%	1.34	0.82	2.07
	Lung and Bronchus	725	249	2.91	2.70	3.13	576	79.4%	5.07	4.67	5.51	149	20.6%	1.10	0.93	1.29
	Cervix Uteri	155	110.29	1.41	1.19	1.64	82	52.9%	1.86	1.48	2.31	73	47.1%	1.10	0.86	1.39
	Kidney and Renal Pelvis	247	180.16	1.37	1.21	1.55	122	49.4%	1.52	1.26	1.82	125	50.6%	1.25	1.04	1.49
	Urinary Bladder	231	137.52	1.68	1.47	1.91	151	65.4%	2.39	2.02	2.80	80	34.6%	1.08	0.85	1.34
	Acute Non-Lymphocytic Leukemia (ANLL)	12	7.89	1.52	0.78	2.66	7	58.3%	2.15	0.86	4.42	5	41.7%	1.08	0.35	2.52
Not Appalachia	**Total Combined 12 Sites**	**4710**	**3091.49**	**1.52**	**1.48**	**1.57**	**3059**	**64.9%**	**1.90**	**1.83**	**1.97**	**1651**	**35.1%**	**1.11**	**1.06**	**1.17**
	Oral Cavity and Pharynx	538	243.95	2.21	2.02	2.40	417	77.5%	3.25	2.95	3.58	121	22.5%	1.05	0.87	1.25
	Esophagus	45	27.49	1.64	1.19	2.19	34	75.6%	2.31	1.60	3.22	11	24.4%	0.86	0.43	1.54
	Stomach	52	38.09	1.37	1.02	1.79	33	63.5%	1.66	1.15	2.34	19	36.5%	1.04	0.63	1.62
	Colon and Rectum	1248	1148.66	1.09	1.03	1.15	697	55.8%	1.17	1.08	1.26	551	44.2%	1.00	0.92	1.08
	Liver	29	19.66	1.48	0.99	2.12	18	62.1%	1.77	1.05	2.79	11	37.9%	1.16	0.58	2.08
	Pancreas	26	18.06	1.44	0.94	2.11	14	53.8%	1.54	0.84	2.58	12	46.2%	1.34	0.69	2.34
	Larynx	248	114.22	2.17	1.91	2.46	194	78.2%	3.16	2.73	3.64	54	21.8%	1.02	0.77	1.33
	Lung and Bronchus	1029	422.13	2.44	2.29	2.59	799	77.6%	3.68	3.43	3.95	230	22.4%	1.12	0.98	1.28
	Cervix Uteri	105	75.6	1.39	1.14	1.68	52	49.5%	1.71	1.28	2.25	53	50.5%	1.17	0.88	1.53
	Kidney and Renal Pelvis	533	405.79	1.31	1.20	1.43	283	53.1%	1.35	1.20	1.51	250	46.9%	1.28	1.12	1.45
	Urinary Bladder	836	561.64	1.49	1.39	1.59	508	60.8%	1.67	1.53	1.82	328	39.2%	1.28	1.14	1.42
	Acute Non-Lymphocytic Leukemia (ANLL)	20	15.71	1.27	0.78	1.97	10	50.0%	1.29	0.62	2.37	10	50.0%	1.26	0.60	2.31
Appalachia	**Total Combined 12 Sites**	**1953**	**1256.26**	**1.55**	**1.49**	**1.63**	**1316**	**67.4%**	**2.02**	**1.91**	**2.13**	**637**	**32.6%**	**1.05**	**0.97**	**1.14**
	Oral Cavity and Pharynx	203	96.68	2.10	1.82	2.41	153	75.4%	3.00	2.54	3.51	50	24.6%	1.10	0.81	1.44
	Esophagus	19	10.12	1.88	1.13	2.93	12	63.2%	2.17	1.12	3.78	7	36.8%	1.53	0.61	3.15
	Stomach	28	21.76	1.29	0.85	1.86	18	64.3%	1.61	0.95	2.54	10	35.7%	0.95	0.45	1.74
	Colon and Rectum	560	481.49	1.16	1.07	1.26	300	53.6%	1.20	1.07	1.35	260	46.4%	1.12	0.99	1.26
	Liver	8	5.84	1.37	0.59	2.70	7	87.5%	2.36	0.94	4.86	1	12.5%	0.35	0.00	1.94
	Pancreas	9	5.98	1.51	0.69	2.86	6	66.7%	2.03	0.74	4.41	3	33.3%	0.99	0.20	2.90
	Larynx	153	56.34	2.72	2.30	3.18	120	78.4%	4.02	3.33	4.81	33	21.6%	1.25	0.86	1.75
	Lung and Bronchus	386	164.63	2.34	2.12	2.59	319	82.6%	3.78	3.37	4.22	67	17.4%	0.84	0.65	1.06
	Cervix Uteri	50	34.69	1.44	1.07	1.90	30	60.0%	2.18	1.47	3.11	20	40.0%	0.96	0.58	1.48
	Kidney and Renal Pelvis	168	148.95	1.13	0.96	1.31	98	58.3%	1.27	1.03	1.55	70	41.7%	0.97	0.76	1.23
	Urinary Bladder	357	225.68	1.58	1.42	1.75	246	68.9%	2.01	1.77	2.28	111	31.1%	1.07	0.88	1.29
	Acute Non-Lymphocytic Leukemia (ANLL)	10	3.92	2.55	1.22	4.69	5	50.0%	2.63	0.85	6.14	5	50.0%	2.48	0.80	5.78

Finally, we evaluated SPC frequency among individuals with other risk factor associated FPCs, including HPV, obesity, physical inactivity, and alcohol related cancers (**Supplementary Table 1**). When combining men and women, survivors of a risk related FPC were at higher risk of any site, same site, and different site SPC than the general population. Risk of same site SPC was also significantly higher than other site cancers for all risk associated cancers evaluated. Alcohol associated (6280) and obesity (6231) were the most common SPC after smoking, with HPV associated cancers conveying the highest risk, SIR 7.62 (95% CI: 3.28-15.0) and SIR 9.78 (95% CI: 7.48-12.6) for men and women, respectively.

Women with a risk factor associated FPC were at increased risk of any, same and other site FPCs when compared to the general population, while men had increased risk of any, same and other alcohol and HPV associated risk. With the exception of alcohol related FPC, there were no differences in SPC risk between men and women. However, men with an alcohol related FPC were at higher risk for an alcohol related SPC than women, SIR 2.29 (95% CI: 2.11-2.48) and SIR 1.61 (95% CI: 1.53-1.68). Similarly, when comparing Appalachian and non-Appalachian populations, both groups had increased risk of any, same and other site SPCs when compared to the general population. With the exception of alcohol related FPC, there were no differences in SPC risk between Appalachian and non-Appalachian residents. However, survivors of an alcohol related FPC living in Appalachia had an increased risk of an alcohol related SPC when compared to non-Appalachian residents, SPC 1.30 (95% CI: 1.22-1.37) and SIR 1.12 (95% CI: 1.08-1.16), respectively.

## 4 Discussion

SPC is a common problem for cancer survivors in Kentucky, with 10.1% of five-year survivors developing one SPC and 1.1% developing two or more in either the same or different sites. When compared to the US population (11), 8.3% of five-year survivors developed a different site SPC, while in our population the risk of different site SPC was 9.2% overall, 9.1% among non-Appalachian Kentuckians and 9.5% among Appalachian Kentuckians. While direct risk comparisons are difficult between Kentucky and US populations since our studies used different denominators (Kentucky general and US general population, respectively), it appears more Kentuckians develop SPC than the individuals residing in other parts of the US. It is also a serious problem, as the majority of survivors diagnosed with a SPC in this study were deceased (55.0%), while only 26% of those without a SPC had died.

Consistent with prior reports suggesting individuals surviving an FPC have higher risks of developing SPCs than the general population, Kentuckians with an FPC are also more likely to develop an SPC. The risk of developing SPCs was significantly higher for 20 of the 30 FPC types in men and for 20 of the 31 FPC types in women. The most common FPC in women with any site SPC were breast, colon and uterine cancers, however, the FPCs with highest risk of a SPC were larynx, lung, and vulva cancers. In men, the most common any site FPCs were prostate, colon and urinary bladder, while an FPC of the oral cavity, larynx or lung conveyed the highest risk of SPC and men with an FPC of prostate were actually less likely to have an SPC. Given that the highest risk FPCs are smoking related, and lung cancer accounts for nearly 25% of all SPC in Kentucky, smoking cessation interventions are highlighted as critical components of survivorship care, as well as careful attention to cancer screening studies (mammography, colonoscopy, CT screening) for survivors of larynx, oral cavity and pharynx cancers

Similarly, SPC frequency among individuals with a risk factor associated FPC, including HPV, obesity, physical inactivity, smoking and alcohol associated cancers was significantly higher than the general population. These effects were especially pronounced in the incidence of same site SPC, notably, male and female survivors of HPV associated FPC had same site SPC excess risks of 762% and 978%, respectively, suggesting that survivors continue high risk behaviors during cancer survivorship and again highlighting opportunities for primary prevention and cancer screening as part of survivorship care. In addition, male and female survivors of HPV associated cancers also had excess SPC of non-risk factor related SPC of 181% and 165%, suggesting factors other than risky behaviors (eg; genetic predisposition, treatment exposure, or environmental exposure) may also contribute to excess risk.

We also demonstrate that cancer survivors residing in Appalachia had a significantly increased risk of SPC compared to cancer survivors residing in non-Appalachian regions. While excess risk in this region has historically been attributed to higher rates of smoking and other cancer risk associated behaviors, in our study cancer survivors residing in Appalachia did not have increased risk of smoking associated SPCs when compared to non-Appalachians. While this could be a function of small sample size for individual cancers or not surviving longer enough (5 years) to be included in the study, genetic and environmental factors could also play a role.

Strengths of this study include a relatively large sample size, use of SEER registry data for the entire state of Kentucky and the inclusion of a Kentucky Appalachian population. This study has several limitations, first, it was conducted in a single state, which provides a robust picture of SPC in Kentucky, but may not be generalizable to other regions. However, given our standing amongst states with the highest incidence and mortality overall from cancer, it represents critical information for Kentucky. Second, only patients surviving more than five years since their first primary cancer diagnosis were included in the data analysis. The approach may provide more stable estimates, however, given the excess mortality and reduced survivorship in the Appalachian region of Kentucky, risk of SPC may have been underestimated. Third, we included same site cancers, and while this increases the risk of bias due to misclassification of recurrence, important insights related to overall excess risk were gained, and we separately reported other site SPC for reference. Fourth, effects of radiation and systemic treatment were not considered due to an inability to determine a temporal relationship between treatments and SPC development. Fifth, survivors migrating out of Kentucky are not reportable and risks may be underestimated, however less than 3% of subjects were lost to follow-up. Sixth, after initial diagnosis, survivors were likely to have increased medical surveillance and an increased likelihood of detection of SPC than the general population, although only SPCs occurring after 5 years were included in an attempt to minimize this bias.

## 5 Conclusions

This is the first report of the increased risk of SPCs among Appalachian cancer survivors when compared to Kentucky cancer survivors, while both are at increased risk when compared to the general Kentucky population. Intriguingly, risk of smoking related SPCs was not different between Appalachian and non-Appalachian survivors, and further study of environmental and genetic risk factors is warranted. Finally, the majority of SPC risk is associated with risk factor associated cancers which highlights important opportunities for primary prevention as part of survivorship care.

## Data availability statement

The raw data supporting the conclusions of this article will be made available by the authors, without undue reservation.

## Ethics statement

The studies involving human participants were reviewed and approved by University of Kentucky Medical Institutional Review Board (IRB). Written informed consent for participation was not required for this study in accordance with the national legislation and the institutional requirements.

## Author contributions

BH, QC, and JK contributed to conception and design of the study. QC performed the statistical analysis. JK wrote the first draft of the manuscript. QC, BH, AA, and ED wrote sections of the manuscript. All authors contributed to the article and approved the submitted version.
