# Supplementary material for: Association of first primary cancer with risk of subsequent primary cancer among survivors of adult-onset cancers in Kentucky and Appalachian Kentucky
Source: Front Oncol. 2023 Aug 17;13:1193487. doi: 10.3389/fonc.2023.1193487 (PMC10470616; doi:10.3389/fonc.2023.1193487)
Supplement: Supplementary file 1 [file DataSheet_1.docx]

Supplementary Material

Association of First Primary Cancer with Risk of Subsequent Primary Cancer Among Survivors of Adult-Onset Cancers in Kentucky and Appalachian Kentucky

Quan Chen, DrPH ^1,2^, Bin Huang, DrPH, MS ^1,2^, Abigail M. Anderson, PhD ^1^, Eric B. Durbin, DrPH, MS ^1,3^, Susanne M. Arnold, MD ^1,5^, Jill M. Kolesar, PharmD, MS ^1,4,6*^

*** Correspondence:** Jill M. Kolesar, PharmD, MS, Department of Pharmacy Practice and Science, College of Pharmacy, University of Kentucky, 760 Press Avenue, Lexington, KY email: jill.kolesar@uky.edu

# Supplementary Data

# Supplementary Figure 1. Top 5 FPC and SPC based on Frequency

#
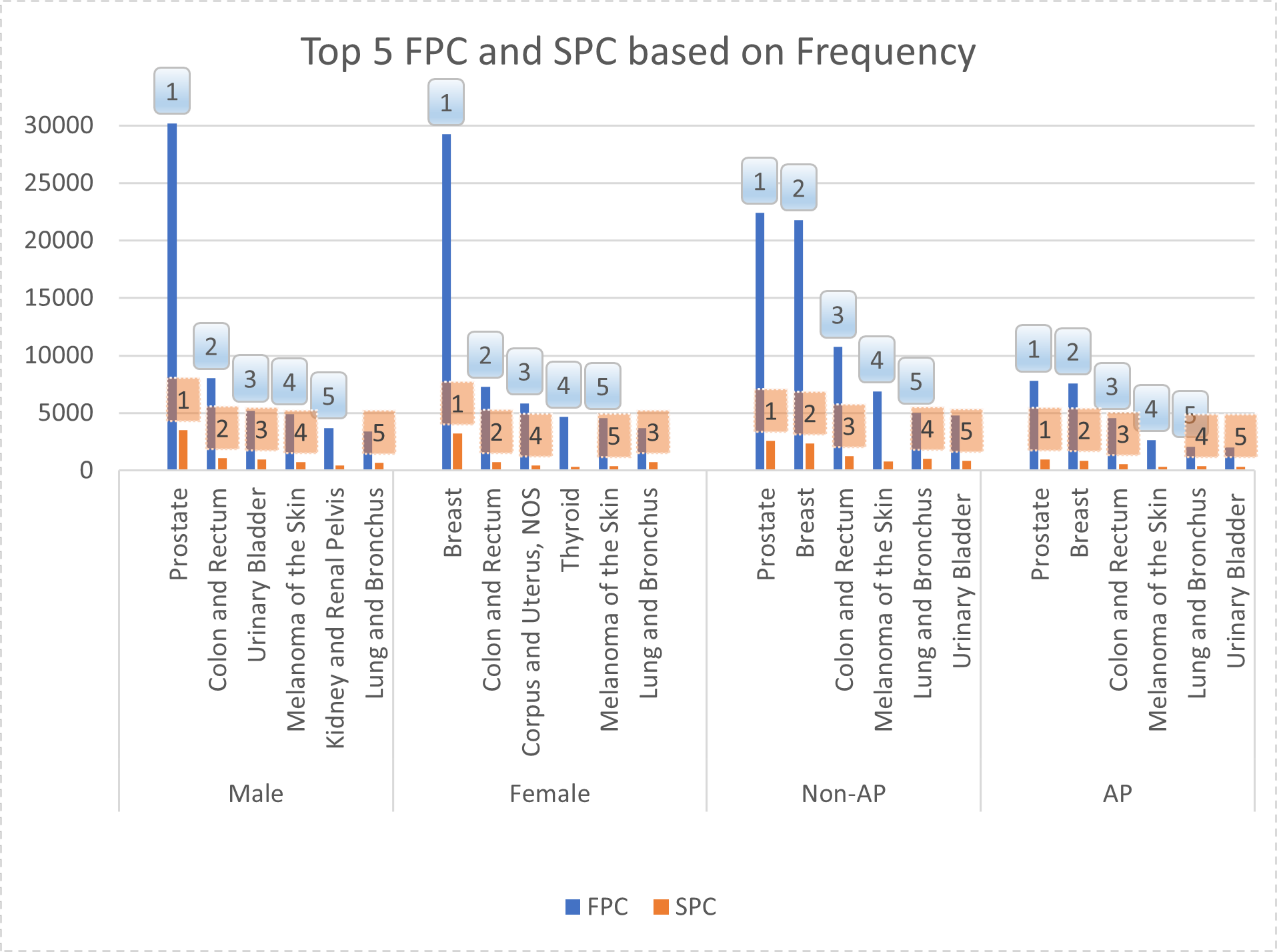


# Supplementary Figure 2. Heatmap of FPC by SPC for male and female.


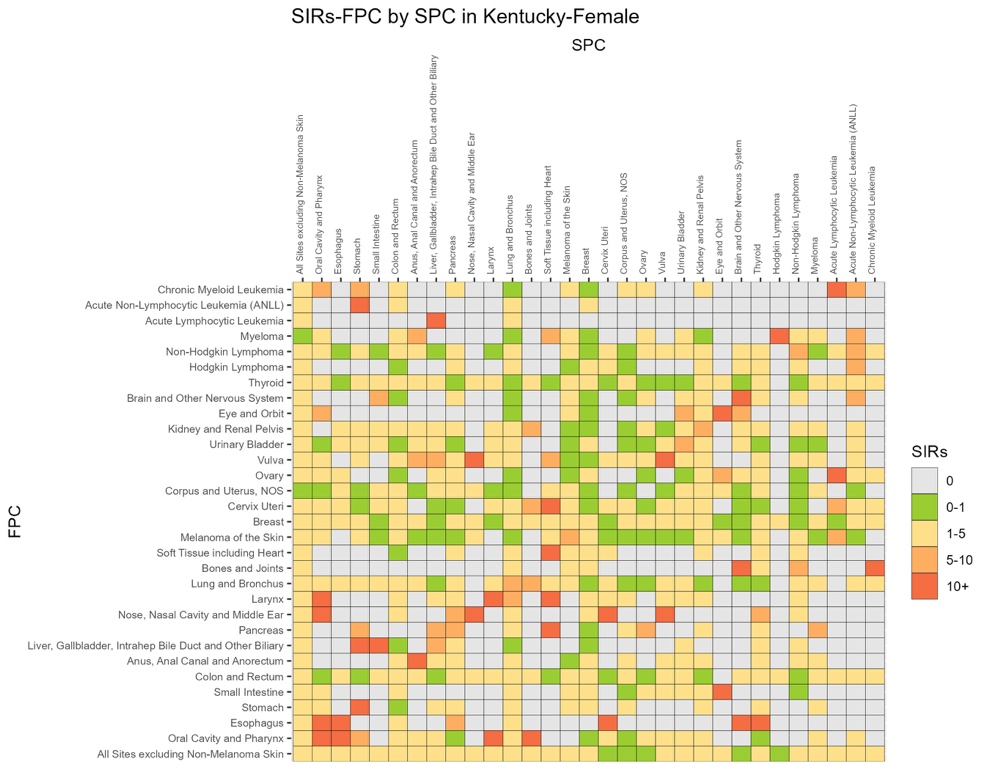

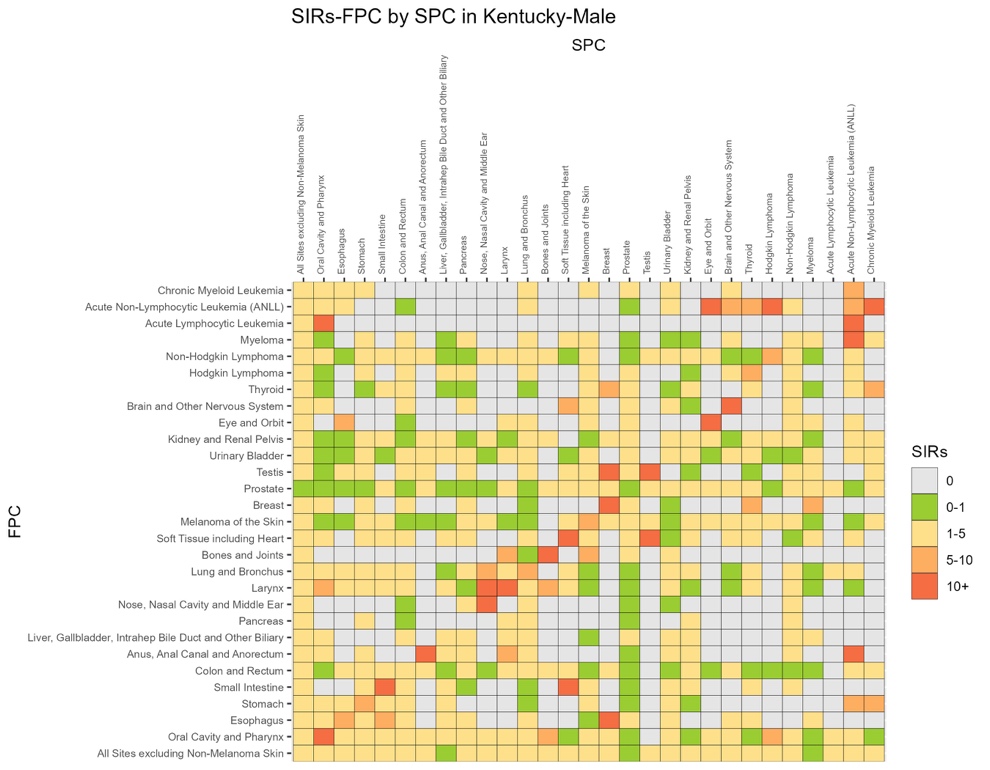


# Supplementary Figure 3. Heatmap of FPC by SPC for Appalachia and Non Appalachia

**
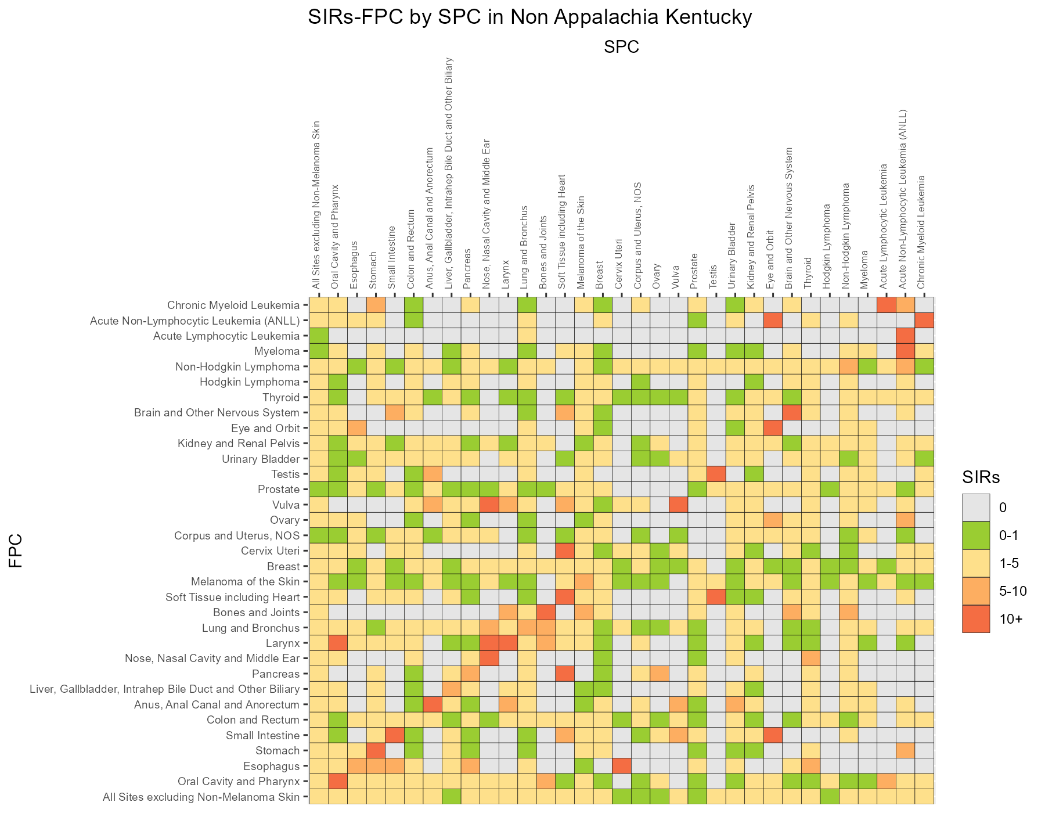
** **
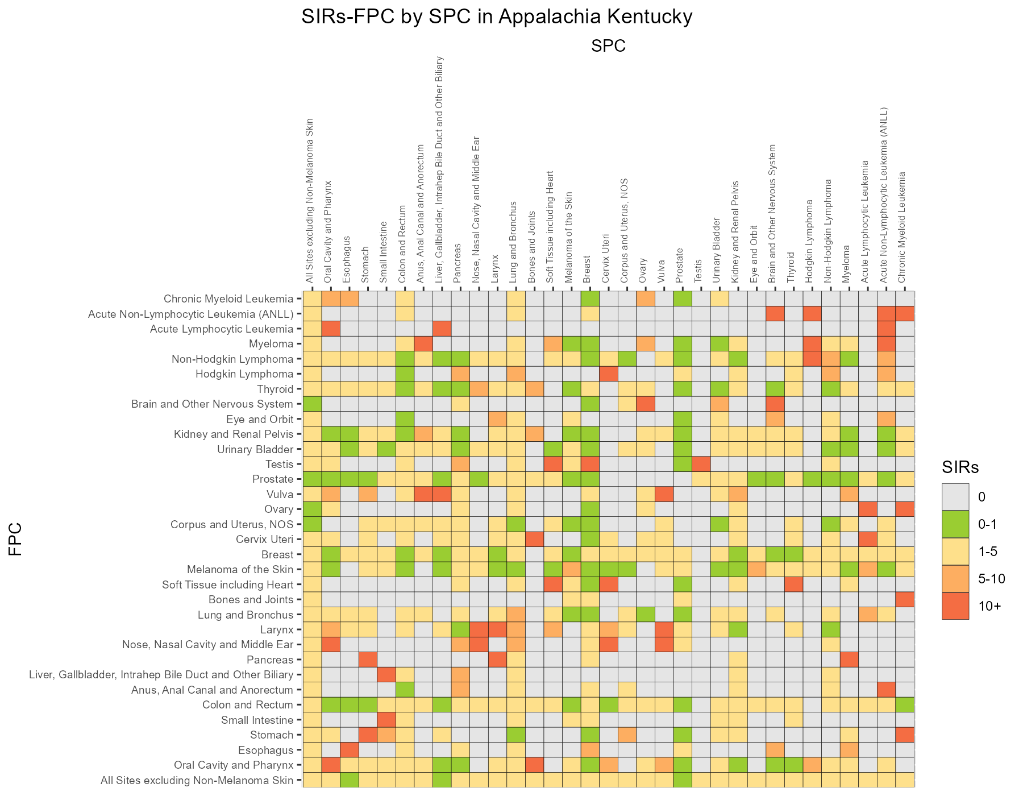
**

# Supplementary Table 1. Other Risk factor related cancers by sex and Appalachia Status

| **HPV** |  |  | **SPC on Any Site** | | | |  | **SPC on Risk Factor Related Sites** | | | | |  | **SPC on non-Risk Factor Related Sites** | | | | |
| --- | --- | --- | --- | --- | --- | --- | --- | --- | --- | --- | --- | --- | --- | --- | --- | --- | --- | --- |
| **Sex** | **FPC Sites** | **Observed SPC** | **Expected SPC** | **SIR** | **CI Lower** | **CI Upper** |  | **Observed SPC** | **%** | **SIR** | **CI Lower** | **CI Upper** |  | **Observed SPC** | **%** | **SIR** | **CI Lower** | **CI Upper** |
| Male and female | Total 6 HPV associated Sites | 666 | 353.88 | 1.88 | 1.74 | 2.03 |  | 69 | 10.4% | 9.47 | 7.36 | 11.98 |  | 597 | 89.6% | 1.72 | 1.59 | 1.87 |
|  | Squamous cell carcinoma of the oropharynx | 291 | 142.35 | 2.04 | 1.82 | 2.29 |  | 3 | 1.0% | 2.14 | 0.43 | 6.26 |  | 288 | 99.0% | 2.04 | 1.81 | 2.29 |
|  | Squamous cell carcinoma of the anus | 94 | 49.61 | 1.89 | 1.53 | 2.32 |  | 4 | 4.3% | 4.26 | 1.14 | 10.89 |  | 90 | 95.7% | 1.85 | 1.49 | 2.27 |
|  | Squamous cell carcinoma of the vulva | 97 | 29.36 | 3.30 | 2.68 | 4.03 |  | 41 | 42.3% | 50.00 | 35.88 | 67.83 |  | 56 | 57.7% | 1.96 | 1.48 | 2.55 |
|  | Squamous cell carcinoma of the vagina | 9 | 5.69 | 1.58 | 0.72 | 3.00 |  | 2 | 22.2% | 14.29 | 1.60 | 51.58 |  | 7 | 77.8% | 1.26 | 0.51 | 2.60 |
|  | Carcinoma of the cervix | 154 | 109.39 | 1.41 | 1.19 | 1.65 |  | 16 | 10.4% | 4.16 | 2.37 | 6.75 |  | 138 | 89.6% | 1.31 | 1.10 | 1.54 |
|  | Squamous cell carcinoma of the penis | 21 | 17.48 | 1.20 | 0.74 | 1.84 |  | 3 | 14.3% | 27.27 | 5.48 | 79.69 |  | 18 | 85.7% | 1.04 | 0.61 | 1.64 |
| Male | Total 6 HPV associated Sites | 289 | 155.9 | 1.85 | 1.65 | 2.08 |  | 8 | 2.8% | 7.62 | 3.28 | 15.01 |  | 281 | 97.2% | 1.81 | 1.61 | 2.04 |
|  | Squamous cell carcinoma of the oropharynx | 236 | 120.5 | 1.96 | 1.72 | 2.22 |  | 3 | 1.3% | 3.66 | 0.74 | 10.69 |  | 233 | 98.7% | 1.95 | 1.70 | 2.21 |
|  | Squamous cell carcinoma of the anus | 32 | 17.92 | 1.79 | 1.22 | 2.52 |  | 2 | 6.3% | 18.18 | 2.04 | 65.65 |  | 30 | 93.8% | 1.68 | 1.14 | 2.40 |
|  | Squamous cell carcinoma of the vulva | 0 | 0 | . | . | . |  | 0 |  | . | . | . |  | 0 |  | . | . | . |
|  | Squamous cell carcinoma of the vagina | 0 | 0 | . | . | . |  | 0 |  | . | . | . |  | 0 |  | . | . | . |
|  | Carcinoma of the cervix | 0 | 0 | . | . | . |  | 0 |  | . | . | . |  | 0 |  | . | . | . |
|  | Squamous cell carcinoma of the penis | 21 | 17.48 | 1.20 | 0.74 | 1.84 |  | 3 | 14.3% | 27.27 | 5.48 | 79.69 |  | 18 | 85.7% | 1.04 | 0.61 | 1.64 |
| Female | Total 6 HPV associated Sites | 377 | 197.98 | 1.90 | 1.72 | 2.11 |  | 61 | 16.2% | 9.78 | 7.48 | 12.56 |  | 316 | 83.8% | 1.65 | 1.47 | 1.84 |
|  | Squamous cell carcinoma of the oropharynx | 55 | 21.85 | 2.52 | 1.90 | 3.28 |  | 0 | 0.0% | 0.00 | . | . |  | 55 | 100.0% | 2.59 | 1.95 | 3.37 |
|  | Squamous cell carcinoma of the anus | 62 | 31.69 | 1.96 | 1.50 | 2.51 |  | 2 | 3.2% | 2.41 | 0.27 | 8.70 |  | 60 | 96.8% | 1.94 | 1.48 | 2.50 |
|  | Squamous cell carcinoma of the vulva | 97 | 29.36 | 3.30 | 2.68 | 4.03 |  | 41 | 42.3% | 50.00 | 35.88 | 67.83 |  | 56 | 57.7% | 1.96 | 1.48 | 2.55 |
|  | Squamous cell carcinoma of the vagina | 9 | 5.69 | 1.58 | 0.72 | 3.00 |  | 2 | 22.2% | 14.29 | 1.60 | 51.58 |  | 7 | 77.8% | 1.26 | 0.51 | 2.60 |
|  | Carcinoma of the cervix | 154 | 109.39 | 1.41 | 1.19 | 1.65 |  | 16 | 10.4% | 4.16 | 2.37 | 6.75 |  | 138 | 89.6% | 1.31 | 1.10 | 1.54 |
|  | Squamous cell carcinoma of the penis | 0 | 0 | . | . | . |  | 0 |  | . | . | . |  | 0 |  | . | . | . |
|  |  |  |  |  |  |  |  |  |  |  |  |  |  |  |  |  |  |  |
| Not Appalachia | Six HPV associated Sites | 471 | 254.8 | 1.85 | 1.69 | 2.02 |  | 45 | 9.6% | 8.70 | 6.35 | 11.65 |  | 426 | 90.4% | 1.71 | 1.55 | 1.88 |
|  | Squamous cell carcinoma of the oropharynx | 210 | 105.74 | 1.99 | 1.73 | 2.27 |  | 2 | 1.0% | 1.94 | 0.22 | 7.01 |  | 208 | 99.0% | 1.99 | 1.73 | 2.28 |
|  | Squamous cell carcinoma of the anus | 81 | 39.07 | 2.07 | 1.65 | 2.58 |  | 4 | 4.9% | 5.41 | 1.45 | 13.84 |  | 77 | 95.1% | 2.01 | 1.59 | 2.51 |
|  | Squamous cell carcinoma of the vulva | 61 | 20.36 | 3.00 | 2.29 | 3.85 |  | 24 | 39.3% | 42.86 | 27.45 | 63.77 |  | 37 | 60.7% | 1.87 | 1.32 | 2.58 |
|  | Squamous cell carcinoma of the vagina | 6 | 4.06 | 1.48 | 0.54 | 3.22 |  | 2 | 33.3% | 20.00 | 2.25 | 72.21 |  | 4 | 66.7% | 1.01 | 0.27 | 2.59 |
|  | Carcinoma of the cervix | 105 | 75.19 | 1.40 | 1.14 | 1.69 |  | 10 | 9.5% | 3.77 | 1.81 | 6.94 |  | 95 | 90.5% | 1.31 | 1.06 | 1.60 |
|  | Squamous cell carcinoma of the penis | 8 | 10.38 | 0.77 | 0.33 | 1.52 |  | 3 | 37.5% | 42.86 | 8.61 | 125.22 |  | 5 | 62.5% | 0.48 | 0.16 | 1.13 |
| Appalachia | Six HPV associated Sites | 195 | 99.08 | 1.97 | 1.70 | 2.26 |  | 24 | 12.3% | 11.27 | 7.22 | 16.77 |  | 171 | 87.7% | 1.76 | 1.51 | 2.05 |
|  | Squamous cell carcinoma of the oropharynx | 81 | 36.61 | 2.21 | 1.76 | 2.75 |  | 1 | 1.2% | 2.86 | 0.04 | 15.90 |  | 80 | 98.8% | 2.21 | 1.75 | 2.75 |
|  | Squamous cell carcinoma of the anus | 13 | 10.54 | 1.23 | 0.66 | 2.11 |  | 0 | 0.0% | 0.00 | . | . |  | 13 | 100.0% | 1.26 | 0.67 | 2.15 |
|  | Squamous cell carcinoma of the vulva | 36 | 9.01 | 4.00 | 2.80 | 5.53 |  | 17 | 47.2% | 68.00 | 39.59 | 108.88 |  | 19 | 52.8% | 2.17 | 1.31 | 3.39 |
|  | Squamous cell carcinoma of the vagina | 3 | 1.62 | 1.85 | 0.37 | 5.41 |  | 0 | 0.0% | 0.00 | . | . |  | 3 | 100.0% | 1.89 | 0.38 | 5.51 |
|  | Carcinoma of the cervix | 49 | 34.2 | 1.43 | 1.06 | 1.89 |  | 6 | 12.2% | 5.00 | 1.83 | 10.88 |  | 43 | 87.8% | 1.30 | 0.94 | 1.76 |
|  | Squamous cell carcinoma of the penis | 13 | 7.1 | 1.83 | 0.97 | 3.13 |  | 0 | 0.0% | 0.00 | . | . |  | 13 | 100.0% | 1.84 | 0.98 | 3.15 |

| **Obesity** |  |  | **SPC on Any Site** | | | |  | **SPC on Risk Factor Related Sites** | | | | |  | **SPC on non-Risk Factor Related Sites** | | | | |
| --- | --- | --- | --- | --- | --- | --- | --- | --- | --- | --- | --- | --- | --- | --- | --- | --- | --- | --- |
| **Sex** | **FPC Sites** | **Observed SPC** | **Expected SPC** | **SIR** | **CI Lower** | **CI Upper** |  | **Observed SPC** | **%** | **SIR** | **CI Lower** | **CI Upper** |  | **Observed SPC** | **%** | **SIR** | **CI Lower** | **CI Upper** |
| Male and female | Total 13 Obesity Sites | 6231 | 5368.87 | 1.16 | 1.13 | 1.19 |  | 3122 | 50.1% | 1.30 | 1.26 | 1.35 |  | 3109 | 49.9% | 1.05 | 1.01 | 1.08 |
|  | Adenocarcinoma of the esophagus | 42 | 29.83 | 1.41 | 1.01 | 1.90 |  | 17 | 40.5% | 2.22 | 1.29 | 3.55 |  | 25 | 59.5% | 1.13 | 0.73 | 1.66 |
|  | Gastric Cardia | 15 | 14.22 | 1.05 | 0.59 | 1.74 |  | 4 | 26.7% | 0.96 | 0.26 | 2.47 |  | 11 | 73.3% | 1.09 | 0.54 | 1.95 |
|  | Colon and Rectum | 1808 | 1630.15 | 1.11 | 1.06 | 1.16 |  | 716 | 39.6% | 1.26 | 1.17 | 1.35 |  | 1092 | 60.4% | 1.03 | 0.97 | 1.09 |
|  | Liver | 37 | 25.5 | 1.45 | 1.02 | 2.00 |  | 13 | 35.1% | 1.53 | 0.81 | 2.62 |  | 24 | 64.9% | 1.41 | 0.90 | 2.10 |
|  | Gallbladder | 8 | 7.76 | 1.03 | 0.44 | 2.03 |  | 3 | 37.5% | 0.94 | 0.19 | 2.76 |  | 5 | 62.5% | 1.09 | 0.35 | 2.55 |
|  | Pancreas | 35 | 24.04 | 1.46 | 1.01 | 2.02 |  | 14 | 40.0% | 1.56 | 0.85 | 2.62 |  | 21 | 60.0% | 1.39 | 0.86 | 2.13 |
|  | Multiple Myeloma | 77 | 79.44 | 0.97 | 0.76 | 1.21 |  | 25 | 32.5% | 0.88 | 0.57 | 1.29 |  | 52 | 67.5% | 1.02 | 0.76 | 1.34 |
|  | Postmenopausal female breast | 2555 | 2067.67 | 1.24 | 1.19 | 1.28 |  | 1511 | 59.1% | 1.38 | 1.32 | 1.46 |  | 1044 | 40.9% | 1.07 | 1.01 | 1.14 |
|  | Corpus and uterus NOS | 450 | 492.56 | 0.91 | 0.83 | 1.00 |  | 274 | 60.9% | 1.03 | 0.91 | 1.16 |  | 176 | 39.1% | 0.78 | 0.67 | 0.90 |
|  | Ovary | 99 | 98.44 | 1.01 | 0.82 | 1.22 |  | 54 | 54.5% | 1.00 | 0.75 | 1.31 |  | 45 | 45.5% | 1.01 | 0.74 | 1.35 |
|  | Kidney | 670 | 533.1 | 1.26 | 1.16 | 1.36 |  | 273 | 40.7% | 1.52 | 1.34 | 1.71 |  | 397 | 59.3% | 1.12 | 1.02 | 1.24 |
|  | Meningioma | 2 | 2.15 | 0.93 | 0.10 | 3.36 |  | 1 | 50.0% | 0.97 | 0.01 | 5.40 |  | 1 | 50.0% | 0.89 | 0.01 | 4.97 |
|  | Thyroid | 433 | 364.01 | 1.19 | 1.08 | 1.31 |  | 217 | 50.1% | 1.25 | 1.09 | 1.42 |  | 216 | 49.9% | 1.14 | 0.99 | 1.30 |
| Male | Total 13 Obesity Sites | 1750 | 1581.61 | 1.11 | 1.06 | 1.16 |  | 504 | 28.8% | 1.35 | 1.23 | 1.47 |  | 1246 | 71.2% | 1.03 | 0.98 | 1.09 |
|  | Adenocarcinoma of the esophagus | 37 | 27.46 | 1.35 | 0.95 | 1.86 |  | 14 | 37.8% | 2.19 | 1.19 | 3.67 |  | 23 | 62.2% | 1.09 | 0.69 | 1.64 |
|  | Gastric Cardia | 12 | 11.75 | 1.02 | 0.53 | 1.78 |  | 4 | 33.3% | 1.44 | 0.39 | 3.70 |  | 8 | 66.7% | 0.89 | 0.38 | 1.76 |
|  | Colon and Rectum | 1056 | 996.95 | 1.06 | 1.00 | 1.13 |  | 300 | 28.4% | 1.28 | 1.14 | 1.44 |  | 756 | 71.6% | 0.99 | 0.92 | 1.06 |
|  | Liver | 32 | 17.98 | 1.78 | 1.22 | 2.51 |  | 11 | 34.4% | 2.49 | 1.24 | 4.46 |  | 21 | 65.6% | 1.55 | 0.96 | 2.37 |
|  | Gallbladder | 1 | 3.06 | 0.33 | 0.00 | 1.82 |  | 0 | 0.0% | 0.00 | . | . |  | 1 | 100.0% | 0.43 | 0.01 | 2.38 |
|  | Pancreas | 14 | 13.63 | 1.03 | 0.56 | 1.72 |  | 3 | 21.4% | 0.92 | 0.18 | 2.68 |  | 11 | 78.6% | 1.06 | 0.53 | 1.90 |
|  | Multiple Myeloma | 49 | 47.21 | 1.04 | 0.77 | 1.37 |  | 12 | 24.5% | 1.08 | 0.56 | 1.88 |  | 37 | 75.5% | 1.03 | 0.72 | 1.41 |
|  | Postmenopausal female breast |  |  |  |  |  |  |  |  |  |  |  |  |  |  |  |  |  |
|  | Corpus and uterus NOS |  |  |  |  |  |  |  |  |  |  |  |  |  |  |  |  |  |
|  | Ovary |  |  |  |  |  |  |  |  |  |  |  |  |  |  |  |  |  |
|  | Kidney | 437 | 360.33 | 1.21 | 1.10 | 1.33 |  | 132 | 30.2% | 1.53 | 1.28 | 1.81 |  | 305 | 69.8% | 1.11 | 0.99 | 1.25 |
|  | Meningioma | 0 | 0.56 | 0.00 | . | . |  | 0 |  | 0.00 | . | . |  | 0 |  | 0.00 | . | . |
|  | Thyroid | 112 | 102.68 | 1.09 | 0.90 | 1.31 |  | 28 | 25.0% | 1.11 | 0.74 | 1.60 |  | 84 | 75.0% | 1.09 | 0.87 | 1.34 |
| Female | Total 13 Obesity Sites | 4481 | 3787.26 | 1.18 | 1.15 | 1.22 |  | 2618 | 58.4% | 1.29 | 1.25 | 1.35 |  | 1863 | 41.6% | 1.06 | 1.01 | 1.10 |
|  | Adenocarcinoma of the esophagus | 5 | 2.37 | 2.11 | 0.68 | 4.92 |  | 3 | 60.0% | 2.40 | 0.48 | 7.01 |  | 2 | 40.0% | 1.79 | 0.20 | 6.45 |
|  | Gastric Cardia | 3 | 2.47 | 1.21 | 0.24 | 3.55 |  | 0 | 0.0% | 0.00 | . | . |  | 3 | 100.0% | 2.73 | 0.55 | 7.97 |
|  | Colon and Rectum | 752 | 633.2 | 1.19 | 1.10 | 1.28 |  | 416 | 55.3% | 1.24 | 1.13 | 1.37 |  | 336 | 44.7% | 1.13 | 1.01 | 1.25 |
|  | Liver | 5 | 7.52 | 0.66 | 0.21 | 1.55 |  | 2 | 40.0% | 0.49 | 0.05 | 1.77 |  | 3 | 60.0% | 0.87 | 0.18 | 2.56 |
|  | Gallbladder | 7 | 4.7 | 1.49 | 0.60 | 3.07 |  | 3 | 42.9% | 1.22 | 0.25 | 3.58 |  | 4 | 57.1% | 1.78 | 0.48 | 4.55 |
|  | Pancreas | 21 | 10.4 | 2.02 | 1.25 | 3.09 |  | 11 | 52.4% | 1.94 | 0.97 | 3.47 |  | 10 | 47.6% | 2.12 | 1.01 | 3.90 |
|  | Multiple Myeloma | 28 | 32.23 | 0.87 | 0.58 | 1.26 |  | 13 | 46.4% | 0.75 | 0.40 | 1.28 |  | 15 | 53.6% | 1.01 | 0.56 | 1.67 |
|  | Postmenopausal female breast | 2555 | 2067.67 | 1.24 | 1.19 | 1.28 |  | 1511 | 59.1% | 1.38 | 1.32 | 1.46 |  | 1044 | 40.9% | 1.07 | 1.01 | 1.14 |
|  | Corpus and uterus NOS | 450 | 492.56 | 0.91 | 0.83 | 1.00 |  | 274 | 60.9% | 1.03 | 0.91 | 1.16 |  | 176 | 39.1% | 0.78 | 0.67 | 0.90 |
|  | Ovary | 99 | 98.44 | 1.01 | 0.82 | 1.22 |  | 54 | 54.5% | 1.00 | 0.75 | 1.31 |  | 45 | 45.5% | 1.01 | 0.74 | 1.35 |
|  | Kidney | 233 | 172.77 | 1.35 | 1.18 | 1.53 |  | 141 | 60.5% | 1.51 | 1.27 | 1.78 |  | 92 | 39.5% | 1.16 | 0.94 | 1.42 |
|  | Meningioma | 2 | 1.59 | 1.26 | 0.14 | 4.54 |  | 1 | 50.0% | 1.11 | 0.01 | 6.18 |  | 1 | 50.0% | 1.45 | 0.02 | 8.06 |
|  | Thyroid | 321 | 261.33 | 1.23 | 1.10 | 1.37 |  | 189 | 58.9% | 1.27 | 1.10 | 1.46 |  | 132 | 41.1% | 1.17 | 0.98 | 1.39 |
|  |  |  |  |  |  |  |  |  |  |  |  |  |  |  |  |  |  |  |
| Not Appalachia | Total 13 Obesity Sites | 4456 | 3858.62 | 1.15 | 1.12 | 1.19 |  | 2267 | 50.9% | 1.31 | 1.26 | 1.37 |  | 2189 | 49.1% | 1.03 | 0.99 | 1.07 |
|  | Adenocarcinoma of the esophagus | 27 | 21.3 | 1.27 | 0.84 | 1.84 |  | 14 | 51.9% | 2.52 | 1.38 | 4.23 |  | 13 | 48.1% | 0.83 | 0.44 | 1.41 |
|  | Gastric Cardia | 8 | 9.66 | 0.83 | 0.36 | 1.63 |  | 2 | 25.0% | 0.72 | 0.08 | 2.62 |  | 6 | 75.0% | 0.87 | 0.32 | 1.89 |
|  | Colon and Rectum | 1248 | 1148.66 | 1.09 | 1.03 | 1.15 |  | 491 | 39.3% | 1.22 | 1.11 | 1.33 |  | 757 | 60.7% | 1.01 | 0.94 | 1.09 |
|  | Liver | 29 | 19.66 | 1.48 | 0.99 | 2.12 |  | 8 | 27.6% | 1.24 | 0.53 | 2.44 |  | 21 | 72.4% | 1.59 | 0.98 | 2.43 |
|  | Gallbladder | 6 | 5.06 | 1.19 | 0.43 | 2.58 |  | 3 | 50.0% | 1.47 | 0.30 | 4.30 |  | 3 | 50.0% | 0.99 | 0.20 | 2.90 |
|  | Pancreas | 26 | 18.06 | 1.44 | 0.94 | 2.11 |  | 10 | 38.5% | 1.50 | 0.72 | 2.76 |  | 16 | 61.5% | 1.40 | 0.80 | 2.28 |
|  | Multiple Myeloma | 53 | 60.05 | 0.88 | 0.66 | 1.15 |  | 18 | 34.0% | 0.83 | 0.49 | 1.30 |  | 35 | 66.0% | 0.92 | 0.64 | 1.27 |
|  | Postmenopausal female breast | 1884 | 1537.81 | 1.23 | 1.17 | 1.28 |  | 1131 | 60.0% | 1.39 | 1.31 | 1.47 |  | 753 | 40.0% | 1.04 | 0.97 | 1.12 |
|  | Corpus and uterus NOS | 311 | 331.99 | 0.94 | 0.84 | 1.05 |  | 183 | 58.8% | 1.02 | 0.88 | 1.18 |  | 128 | 41.2% | 0.84 | 0.70 | 1.00 |
|  | Ovary | 70 | 68.77 | 1.02 | 0.79 | 1.29 |  | 43 | 61.4% | 1.14 | 0.83 | 1.54 |  | 27 | 38.6% | 0.87 | 0.57 | 1.26 |
|  | Kidney | 509 | 391.03 | 1.30 | 1.19 | 1.42 |  | 219 | 43.0% | 1.65 | 1.44 | 1.89 |  | 290 | 57.0% | 1.12 | 1.00 | 1.26 |
|  | Meningioma | 2 | 1.8 | 1.11 | 0.12 | 4.01 |  | 1 | 50.0% | 1.23 | 0.02 | 6.87 |  | 1 | 50.0% | 1.01 | 0.01 | 5.62 |
|  | Thyroid | 283 | 244.76 | 1.16 | 1.03 | 1.30 |  | 144 | 50.9% | 1.23 | 1.04 | 1.45 |  | 139 | 49.1% | 1.09 | 0.92 | 1.29 |
| Appalachia | Total 13 Obesity Sites | 1775 | 1510.25 | 1.18 | 1.12 | 1.23 |  | 855 | 48.2% | 1.28 | 1.20 | 1.37 |  | 920 | 51.8% | 1.09 | 1.02 | 1.16 |
|  | Adenocarcinoma of the esophagus | 15 | 8.53 | 1.76 | 0.98 | 2.90 |  | 3 | 20.0% | 1.43 | 0.29 | 4.17 |  | 12 | 80.0% | 1.87 | 0.96 | 3.26 |
|  | Gastric Cardia | 7 | 4.56 | 1.54 | 0.61 | 3.16 |  | 2 | 28.6% | 1.47 | 0.17 | 5.31 |  | 5 | 71.4% | 1.56 | 0.50 | 3.65 |
|  | Colon and Rectum | 560 | 481.49 | 1.16 | 1.07 | 1.26 |  | 225 | 40.2% | 1.35 | 1.18 | 1.54 |  | 335 | 59.8% | 1.06 | 0.95 | 1.18 |
|  | Liver | 8 | 5.84 | 1.37 | 0.59 | 2.70 |  | 5 | 62.5% | 2.46 | 0.79 | 5.75 |  | 3 | 37.5% | 0.79 | 0.16 | 2.30 |
|  | Gallbladder | 2 | 2.7 | 0.74 | 0.08 | 2.67 |  | 0 | 0.0% | 0.00 | . | . |  | 2 | 100.0% | 1.27 | 0.14 | 4.57 |
|  | Pancreas | 9 | 5.98 | 1.51 | 0.69 | 2.86 |  | 4 | 44.4% | 1.75 | 0.47 | 4.49 |  | 5 | 55.6% | 1.35 | 0.44 | 3.15 |
|  | Multiple Myeloma | 24 | 19.39 | 1.24 | 0.79 | 1.84 |  | 7 | 29.2% | 1.05 | 0.42 | 2.16 |  | 17 | 70.8% | 1.34 | 0.78 | 2.14 |
|  | Postmenopausal female breast | 671 | 529.86 | 1.27 | 1.17 | 1.37 |  | 380 | 56.6% | 1.37 | 1.23 | 1.51 |  | 291 | 43.4% | 1.16 | 1.03 | 1.30 |
|  | Corpus and uterus NOS | 139 | 160.57 | 0.87 | 0.73 | 1.02 |  | 91 | 65.5% | 1.05 | 0.85 | 1.29 |  | 48 | 34.5% | 0.65 | 0.48 | 0.86 |
|  | Ovary | 29 | 29.67 | 0.98 | 0.65 | 1.40 |  | 11 | 37.9% | 0.68 | 0.34 | 1.21 |  | 18 | 62.1% | 1.34 | 0.79 | 2.12 |
|  | Kidney | 161 | 142.08 | 1.13 | 0.96 | 1.32 |  | 54 | 33.5% | 1.14 | 0.86 | 1.49 |  | 107 | 66.5% | 1.13 | 0.92 | 1.36 |
|  | Meningioma | 0 | 0.35 | 0.00 | . | . |  |  |  |  |  |  |  |  |  |  | . | . |
|  | Thyroid | 150 | 119.25 | 1.26 | 1.06 | 1.48 |  | 73 | 48.7% | 1.28 | 1.01 | 1.61 |  | 77 | 51.3% | 1.23 | 0.97 | 1.54 |

| **Physical Inactivity** |  |  | **SPC on Any Site** | | | |  | **SPC on Risk Factor Related Sites** | | | | |  | **SPC on non-Risk Factor Related Sites** | | | | |
| --- | --- | --- | --- | --- | --- | --- | --- | --- | --- | --- | --- | --- | --- | --- | --- | --- | --- | --- |
| **Sex** | **FPC Sites** | **Observed SPC** | **Expected SPC** | **SIR** | **CI Lower** | **CI Upper** |  | **Observed SPC** | **%** | **SIR** | **CI Lower** | **CI Upper** |  | **Observed SPC** | **%** | **SIR** | **CI Lower** | **CI Upper** |
| Male and female | **Combined** | 4221 | 3671.12 | 1.15 | 1.12 | 1.19 |  | 1622 | 38.4% | 1.39 | 1.33 | 1.46 |  | 2599 | 61.6% | 1.04 | 1.00 | 1.08 |
|  | Postmenopausal female Breast | 2555 | 2067.67 | 1.24 | 1.19 | 1.28 |  | 1163 | 45.5% | 1.52 | 1.43 | 1.61 |  | 1392 | 54.5% | 1.07 | 1.01 | 1.13 |
|  | Corpus and uterus NOS | 450 | 492.56 | 0.91 | 0.83 | 1.00 |  | 183 | 40.7% | 0.98 | 0.84 | 1.13 |  | 267 | 59.3% | 0.88 | 0.77 | 0.99 |
|  | Colon | 1216 | 1110.88 | 1.09 | 1.03 | 1.16 |  | 276 | 22.7% | 1.31 | 1.16 | 1.48 |  | 940 | 77.3% | 1.04 | 0.98 | 1.11 |
| Male | **Combined** | 699 | 661.95 | 1.06 | 0.98 | 1.14 |  | 77 | 11.0% | 1.66 | 1.31 | 2.07 |  | 622 | 89.0% | 1.01 | 0.93 | 1.09 |
|  | Postmenopausal female Breast |  |  |  |  |  |  |  |  |  |  |  |  |  |  |  |  |  |
|  | Corpus and uterus NOS |  |  |  |  |  |  |  |  |  |  |  |  |  |  |  |  |  |
|  | Colon | 699 | 661.95 | 1.06 | 0.98 | 1.14 |  | 77 | 11.0% | 1.66 | 1.31 | 2.07 |  | 622 | 89.0% | 1.01 | 0.93 | 1.09 |
| Female | **Combined** | 3522 | 3009.17 | 1.17 | 1.13 | 1.21 |  | 1545 | 43.9% | 1.38 | 1.31 | 1.45 |  | 1977 | 56.1% | 1.05 | 1.00 | 1.09 |
|  | Postmenopausal female Breast | 2555 | 2067.67 | 1.24 | 1.19 | 1.28 |  | 1163 | 45.5% | 1.52 | 1.43 | 1.61 |  | 1392 | 54.5% | 1.07 | 1.01 | 1.13 |
|  | Corpus and uterus NOS | 450 | 492.56 | 0.91 | 0.83 | 1.00 |  | 183 | 40.7% | 0.98 | 0.84 | 1.13 |  | 267 | 59.3% | 0.88 | 0.77 | 0.99 |
|  | Colon | 517 | 448.94 | 1.15 | 1.05 | 1.26 |  | 199 | 38.5% | 1.21 | 1.05 | 1.40 |  | 318 | 61.5% | 1.12 | 1.00 | 1.24 |
|  |  |  |  |  |  |  |  |  |  |  |  |  |  |  |  |  |  |  |
|  |  |  |  |  |  |  |  |  |  |  |  |  |  |  |  |  |  |  |
| Not Appalachia | Combined | 3044 | 2651.53 | 1.15 | 1.11 | 1.19 |  | 1198 | 39.4% | 1.42 | 1.34 | 1.50 |  | 1846 | 60.6% | 1.02 | 0.98 | 1.07 |
|  | Postmenopausal female Breast | 1884 | 1537.81 | 1.23 | 1.17 | 1.28 |  | 870 | 46.2% | 1.53 | 1.43 | 1.63 |  | 1014 | 53.8% | 1.05 | 0.98 | 1.11 |
|  | Corpus and uterus NOS | 311 | 331.99 | 0.94 | 0.84 | 1.05 |  | 125 | 40.2% | 0.99 | 0.82 | 1.18 |  | 186 | 59.8% | 0.90 | 0.78 | 1.04 |
|  | Colon | 849 | 781.72 | 1.09 | 1.01 | 1.16 |  | 203 | 23.9% | 1.36 | 1.18 | 1.56 |  | 646 | 76.1% | 1.02 | 0.94 | 1.10 |
| Appalachia | Combined | 1177 | 1019.59 | 1.15 | 1.09 | 1.22 |  | 424 | 36.0% | 1.33 | 1.21 | 1.46 |  | 753 | 64.0% | 1.07 | 1.00 | 1.15 |
|  | Postmenopausal female Breast | 671 | 529.86 | 1.27 | 1.17 | 1.37 |  | 293 | 43.7% | 1.50 | 1.33 | 1.68 |  | 378 | 56.3% | 1.13 | 1.02 | 1.25 |
|  | Corpus and uterus NOS | 139 | 160.57 | 0.87 | 0.73 | 1.02 |  | 58 | 41.7% | 0.95 | 0.72 | 1.22 |  | 81 | 58.3% | 0.82 | 0.65 | 1.01 |
|  | Colon | 367 | 329.16 | 1.11 | 1.00 | 1.24 |  | 73 | 19.9% | 1.19 | 0.94 | 1.50 |  | 294 | 80.1% | 1.10 | 0.98 | 1.23 |

| **Alcohol** |  |  | **SPC on Any Site** | | | |  | **SPC on Risk Factor Related Sites** | | | | |  | **SPC on non-Risk Factor Related Sites** | | | | |
| --- | --- | --- | --- | --- | --- | --- | --- | --- | --- | --- | --- | --- | --- | --- | --- | --- | --- | --- |
| **Sex** | **FPC Sites** | **Observed SPC** | **Expected SPC** | **SIR** | **CI Lower** | **CI Upper** |  | **Observed SPC** | **%** | **SIR** | **CI Lower** | **CI Upper** |  | **Observed SPC** | **%** | **SIR** | **CI Lower** | **CI Upper** |
| Male and female | Total 6 Alcohol Associated | 6280 | 4674.14 | 1.34 | 1.31 | 1.38 |  | 2546 | 40.5% | 1.72 | 1.66 | 1.79 |  | 3734 | 59.5% | 1.17 | 1.13 | 1.21 |
|  | Oral Cavity and Pharynx | 741 | 340.63 | 2.18 | 2.02 | 2.34 |  | 331 | 44.7% | 4.29 | 3.84 | 4.78 |  | 410 | 55.3% | 1.56 | 1.41 | 1.71 |
|  | Esophagus | 64 | 37.61 | 1.70 | 1.31 | 2.17 |  | 17 | 26.6% | 2.22 | 1.29 | 3.56 |  | 47 | 73.4% | 1.57 | 1.15 | 2.09 |
|  | Colon and Rectum | 1808 | 1630.15 | 1.11 | 1.06 | 1.16 |  | 526 | 29.1% | 1.28 | 1.17 | 1.40 |  | 1282 | 70.9% | 1.05 | 0.99 | 1.11 |
|  | Liver | 37 | 25.5 | 1.45 | 1.02 | 2.00 |  | 11 | 29.7% | 1.76 | 0.88 | 3.15 |  | 26 | 70.3% | 1.35 | 0.88 | 1.98 |
|  | Larynx | 401 | 170.56 | 2.35 | 2.13 | 2.59 |  | 127 | 31.7% | 3.56 | 2.96 | 4.23 |  | 274 | 68.3% | 2.03 | 1.80 | 2.29 |
|  | Breast | 3229 | 2469.7 | 1.31 | 1.26 | 1.35 |  | 1534 | 47.5% | 1.63 | 1.55 | 1.72 |  | 1695 | 52.5% | 1.11 | 1.06 | 1.16 |
| Male | Total 6 Alcohol Associated | 2038 | 1474 | 1.38 | 1.32 | 1.44 |  | 593 | 29.1% | 2.29 | 2.11 | 2.48 |  | 1445 | 70.9% | 1.19 | 1.13 | 1.25 |
|  | Oral Cavity and Pharynx | 569 | 265.6 | 2.14 | 1.97 | 2.33 |  | 237 | 41.7% | 4.88 | 4.28 | 5.55 |  | 332 | 58.3% | 1.53 | 1.37 | 1.70 |
|  | Esophagus | 50 | 32.6 | 1.53 | 1.14 | 2.02 |  | 11 | 22.0% | 1.91 | 0.95 | 3.42 |  | 39 | 78.0% | 1.45 | 1.03 | 1.99 |
|  | Colon and Rectum | 1056 | 996.95 | 1.06 | 1.00 | 1.13 |  | 234 | 22.2% | 1.35 | 1.18 | 1.53 |  | 822 | 77.8% | 1.00 | 0.93 | 1.07 |
|  | Liver | 32 | 17.98 | 1.78 | 1.22 | 2.51 |  | 10 | 31.3% | 2.96 | 1.42 | 5.44 |  | 22 | 68.8% | 1.51 | 0.94 | 2.28 |
|  | Larynx | 305 | 144.06 | 2.12 | 1.89 | 2.37 |  | 93 | 30.5% | 3.65 | 2.94 | 4.47 |  | 212 | 69.5% | 1.79 | 1.56 | 2.05 |
|  | Breast | 26 | 16.81 | 1.55 | 1.01 | 2.27 |  | 8 | 30.8% | 2.69 | 1.16 | 5.31 |  | 18 | 69.2% | 1.30 | 0.77 | 2.06 |
| Female | Total 6 Alcohol Associated | 4242 | 3200.14 | 1.33 | 1.29 | 1.37 |  | 1953 | 46.0% | 1.61 | 1.53 | 1.68 |  | 2289 | 54.0% | 1.15 | 1.11 | 1.20 |
|  | Oral Cavity and Pharynx | 172 | 75.02 | 2.29 | 1.96 | 2.66 |  | 94 | 54.7% | 3.29 | 2.66 | 4.02 |  | 78 | 45.3% | 1.68 | 1.33 | 2.10 |
|  | Esophagus | 14 | 5.01 | 2.79 | 1.53 | 4.69 |  | 6 | 42.9% | 3.19 | 1.17 | 6.95 |  | 8 | 57.1% | 2.56 | 1.10 | 5.04 |
|  | Colon and Rectum | 752 | 633.2 | 1.19 | 1.10 | 1.28 |  | 292 | 38.8% | 1.23 | 1.09 | 1.38 |  | 460 | 61.2% | 1.16 | 1.06 | 1.27 |
|  | Liver | 5 | 7.52 | 0.66 | 0.21 | 1.55 |  | 1 | 20.0% | 0.35 | 0.00 | 1.94 |  | 4 | 80.0% | 0.86 | 0.23 | 2.20 |
|  | Larynx | 96 | 26.5 | 3.62 | 2.93 | 4.42 |  | 34 | 35.4% | 3.32 | 2.30 | 4.64 |  | 62 | 64.6% | 3.81 | 2.92 | 4.89 |
|  | Breast | 3203 | 2452.88 | 1.31 | 1.26 | 1.35 |  | 1526 | 47.6% | 1.63 | 1.55 | 1.71 |  | 1677 | 52.4% | 1.11 | 1.05 | 1.16 |
|  |  |  |  |  |  |  |  |  |  |  |  |  |  |  |  |  |  |  |
| Not Appalachia | Total 6 Alcohol Associated | 4461 | 3389.67 | 1.32 | 1.28 | 1.36 |  | 1876 | 42.1% | 1.74 | 1.66 | 1.82 |  | 2585 | 57.9% | 1.12 | 1.08 | 1.16 |
|  | Oral Cavity and Pharynx | 538 | 243.95 | 2.21 | 2.02 | 2.40 |  | 247 | 45.9% | 4.45 | 3.91 | 5.04 |  | 291 | 54.1% | 1.54 | 1.37 | 1.73 |
|  | Esophagus | 45 | 27.49 | 1.64 | 1.19 | 2.19 |  | 13 | 28.9% | 2.27 | 1.21 | 3.88 |  | 32 | 71.1% | 1.47 | 1.01 | 2.08 |
|  | Colon and Rectum | 1248 | 1148.66 | 1.09 | 1.03 | 1.15 |  | 371 | 29.7% | 1.28 | 1.15 | 1.42 |  | 877 | 70.3% | 1.02 | 0.95 | 1.09 |
|  | Liver | 29 | 19.66 | 1.48 | 0.99 | 2.12 |  | 8 | 27.6% | 1.68 | 0.73 | 3.32 |  | 21 | 72.4% | 1.41 | 0.87 | 2.15 |
|  | Larynx | 248 | 114.22 | 2.17 | 1.91 | 2.46 |  | 87 | 35.1% | 3.69 | 2.95 | 4.55 |  | 161 | 64.9% | 1.78 | 1.51 | 2.07 |
|  | Breast | 2353 | 1835.69 | 1.28 | 1.23 | 1.33 |  | 1150 | 48.9% | 1.65 | 1.55 | 1.74 |  | 1203 | 51.1% | 1.06 | 1.00 | 1.12 |
| Appalachia | Total 6 Alcohol Associated | 1819 | 1284.47 | 1.42 | 1.35 | 1.48 |  | 670 | 36.8% | 1.68 | 1.56 | 1.82 |  | 1149 | 63.2% | 1.30 | 1.22 | 1.37 |
|  | Oral Cavity and Pharynx | 203 | 96.68 | 2.10 | 1.82 | 2.41 |  | 84 | 41.4% | 3.89 | 3.10 | 4.81 |  | 119 | 58.6% | 1.58 | 1.31 | 1.90 |
|  | Esophagus | 19 | 10.12 | 1.88 | 1.13 | 2.93 |  | 4 | 21.1% | 2.11 | 0.57 | 5.39 |  | 15 | 78.9% | 1.82 | 1.02 | 3.01 |
|  | Colon and Rectum | 560 | 481.49 | 1.16 | 1.07 | 1.26 |  | 155 | 27.7% | 1.29 | 1.09 | 1.51 |  | 405 | 72.3% | 1.12 | 1.02 | 1.24 |
|  | Liver | 8 | 5.84 | 1.37 | 0.59 | 2.70 |  | 3 | 37.5% | 2.00 | 0.40 | 5.84 |  | 5 | 62.5% | 1.15 | 0.37 | 2.69 |
|  | Larynx | 153 | 56.34 | 2.72 | 2.30 | 3.18 |  | 40 | 26.1% | 3.31 | 2.36 | 4.50 |  | 113 | 73.9% | 2.55 | 2.11 | 3.07 |
|  | Breast | 876 | 634 | 1.38 | 1.29 | 1.48 |  | 384 | 43.8% | 1.60 | 1.44 | 1.77 |  | 492 | 56.2% | 1.25 | 1.14 | 1.37 |

**
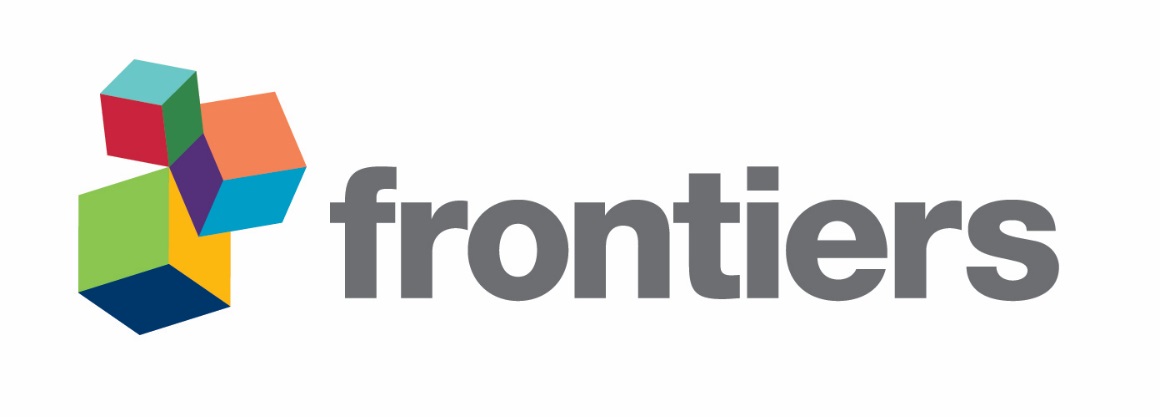
**
